# A novel small molecule chaperone of rod opsin and its potential therapy for retinal degeneration

**DOI:** 10.1038/s41467-018-04261-1

**Published:** 2018-05-17

**Authors:** Yuanyuan Chen, Yu Chen, Beata Jastrzebska, Marcin Golczak, Sahil Gulati, Hong Tang, William Seibel, Xiaoyu Li, Hui Jin, Yong Han, Songqi Gao, Jianye Zhang, Xujie Liu, Hossein Heidari-Torkabadi, Phoebe L. Stewart, William E. Harte, Gregory P. Tochtrop, Krzysztof Palczewski

**Affiliations:** 1 Department of Pharmacology, School of Medicine, Case Western Reserve University, 10900 Euclid Avenue, Cleveland, OH 44106 USA; 2 The McGowan Institute for Regenerative Medicine, University of Pittsburgh, 450 Technology Drive Suite 300, Pittsburgh, PA 15219 USA; 3 Cleveland Center for Membrane and Structural Biology, Case Western Reserve University, 1819 E. 101st Street, Cleveland, OH 44106 USA; 4 Drug Discovery Center, University of Cincinnati, 2180 E. Galbraith Road, Cincinnati, OH 45237 USA; 5 Department of Chemistry, Case Western Reserve University, 10900 Euclid Avenue, Cleveland, OH 44106 USA; 6 Office of Translation and Innovation, Case Western Reserve University, 10900 Euclid Avenue, Cleveland, OH 44106 USA; 7 Present Address: Department of Ophthalmology, University of Pittsburgh, 3501 Fifth Avenue, Pittsburgh, PA 15260 USA; 8 Present Address: Yueyang Hospital and Clinical Research Institute of Integrative Medicine, Shanghai University of Traditional Chinese Medicine, 200437 Shanghai, China

## Abstract

Rhodopsin homeostasis is tightly coupled to rod photoreceptor cell survival and vision. Mutations resulting in the misfolding of rhodopsin can lead to autosomal dominant retinitis pigmentosa (adRP), a progressive retinal degeneration that currently is untreatable. Using a cell-based high-throughput screen (HTS) to identify small molecules that can stabilize the P23H-opsin mutant, which causes most cases of adRP, we identified a novel pharmacological chaperone of rod photoreceptor opsin, YC-001. As a non-retinoid molecule, YC-001 demonstrates micromolar potency and efficacy greater than 9-*cis*-retinal with lower cytotoxicity. YC-001 binds to bovine rod opsin with an EC_50_ similar to 9-*cis*-retinal. The chaperone activity of YC-001 is evidenced by its ability to rescue the transport of multiple rod opsin mutants in mammalian cells. YC-001 is also an inverse agonist that non-competitively antagonizes rod opsin signaling. Significantly, a single dose of YC-001 protects *Abca4*^*−/−*^*Rdh8*^*−/−*^ mice from bright light-induced retinal degeneration, suggesting its broad therapeutic potential.

## Abstract (web summary)

Mutations that lead to misfolding of rhodopsin can cause retinitis pigmentosa. Here, the authors carry out a high throughput screen to identify a small molecule chaperone of rod opsin, and show that it protects mouse models of retinitis pigmentosa from retinal degeneration.

## Introduction

Protein misfolding diseases, collectively referred to as proteopathies, are associated with a variety of neurodegenerative, metabolic, and muscular conditions, as well as visual disorders^1^. Mutations destabilizing RPE65 (retinoid isomerase), ATP-binding cassette subfamily A member 4 (ABCA4), or rhodopsin (rod visual pigment) are associated with inherited retinal degenerations including Leber congenital amaurosis^2,3^, Stargardt disease^4,5^, or adRP^6,7^, respectively (RetNet, http://www.sph.uth.tmc.edu/RetNet/). Unfortunately, most inherited retinal degenerations currently lack effective treatments.

The P23H rhodopsin mutation represents the most common mutation among autosomal dominant retinitis pigmentosa (adRP) patients in North America^8^. This single mutation is an example of class II rhodopsin mutations that share common features indicating the structural instability of rod opsin^9–11^. Owing to its inherent instability, most of P23H rhodopsin undergoes endoplasmic reticulum (ER)-associated protein degradation (ERAD)^10,12^ but small amounts of this mutant rhodopsin escape the ER and cause aberrant rod outer segment (ROS) disc organization and progressive rod photoreceptor cell death^13–16^. In mammalian cells, the P23H rod opsin accumulates in the ER, as manifested by immature glycosylation^9,17,18^. Therefore, we hypothesized that stabilizing P23H rhodopsin could help restore rhodopsin homeostasis and prevent photoreceptor cell death.

**Fig. 1:**
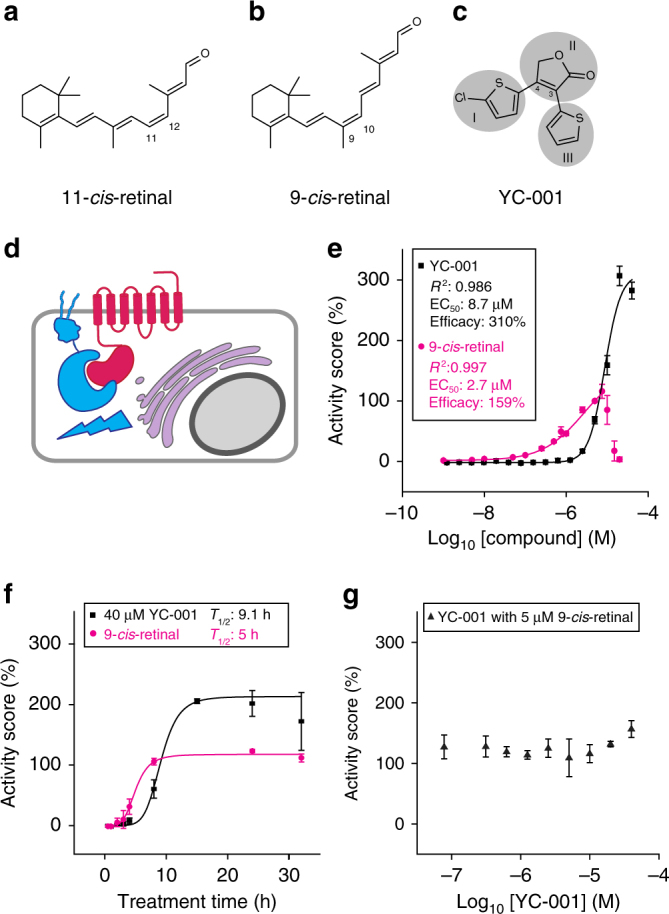
YC-001 rescues P23H opsin from the ER to the plasma membrane. **a**–**c** Chemical structures of 11-*cis*-retinal, 9-*cis*-retinal, and YC-001, respectively. The three chemical moieties of YC-001 are shaded and numbered. **d**. Diagram of the β-Gal fragment complementation assay used for the HTS. Briefly, two complementary fragments of β-Gal (EA and PK) were individually fused with a plasma membrane-anchored peptide, the pleckstrin homology domain of phospholipase C δ (PLC-EA, in cyan), and the mouse P23H-opsin mutant (P23H-PK, in magenta), respectively. A U2OS stable cell line was generated that co-expressed both PLC-EA and P23H-PK. Owing to its inherent instability, P23H-PK accumulated in the ER, whereas PLC-EA remained on the plasma membrane, leading to a loss of β-Gal activity due to the separation of the two fragments of this enzyme. Upon treatment with an active compound that rescues the folding and transport of P23H opsin to the plasma membrane, a recovery of β-Gal activity is observed due to co-localization of PK and EA. **e** The activities of YC-001 (black boxes) and 9-*cis*-retinal (magenta circles) were tested in a dose-dependent manner employing the β-Gal fragment complementation assay. Each compound was preincubated for 24 h before β-Gal activity was tested. Activity scores were standardized to the effect of 5 µM 9-*cis*-retinal as 100%. Dose dependence was fitted by the Hill function with Origin software. *R*^2^, EC_50_ (μM), and Max score for each compound were obtained from curve fitting and are listed in the graph. The experiment was repeated three times. **f** Activities of 40 µM YC-001 (black boxes) and 5 µM 9-*cis*-retinal (magenta circles) were tested as a function of time with the β-Gal fragment complementation assay. The time course graph was fitted with a Hill function and *T*_1/2_s were obtained and listed in the graph. This experiment was repeated twice. **g** Activities of YC-001 together with 5 µM 9-*cis*-retinal were tested in a dose-dependent manner and plotted in black triangles. This experiment was repeated twice. The activity scores were plotted as the averages of three biological replicates, with the error bars as the s.d.s

In many cases, the natural ligand of a G protein-coupled receptor (GPCR) also promotes its folding by shifting the energy balance towards its native conformation. For example, the visual chromophore, 11-*cis*-retinal, or its analog 9-*cis*-retinal (Fig. 1a, b), stabilizes both wild-type (WT) and P23H rod opsin and improves their biosynthesis and transport^18,19^. Functional rescue of GPCRs carrying folding defects with pharmacological chaperons is also observed for the arginine vasopressin receptor 2^20^, opioid^21^, luteinizing hormone^22^, β1-adrenergic^23^, and Frizzled4 receptors^24^, among others.

Rhodopsin, a highly expressed rod photoreceptor visual pigment protein, is a GPCR activated by a photon that initiates a cascade of intracellular responses eventually leading to visual perception^25,26^. Owing to its abundance and essential physiological function, a pharmacological chaperone and modulator of rod opsin could slow the visual cycle for treatment of retinal degenerations related to all-*trans*-retinal toxicity, such as Stargardt disease^27–29^, and adRP resulting from rhodopsin folding defects^30^. However, compared to other GPCRs, little is known about small-molecule non-retinoid modulators of rod opsin other than 11-*cis*-retinal or its analogs, because photoreceptor-specific signaling pathways are difficult to reproduce in mammalian cell cultures for small-molecule screening.

Here, our effort to identify small molecules that rescue the transport of P23H rod opsin led to the identification of a novel pharmacological chaperone of rod opsin, YC-001, which showed inverse agonist and non-competitive antagonist activities towards rod opsin. Just one preconditioning dose of YC-001 protected *Abca4*^*−/−*^*Rdh8*^*−/−*^ mice from bright light-induced photoreceptor death, suggesting its broad application against retinal degeneration.

## Results

### Identification of YC-001 by high-throughput screen (HTS)

Using a cell-based β-Gal fragment complementation assay, a HTS was carried out to identify small molecules that promote the transport of the unstable P23H-mutant opsin protein from the ER to the plasma membrane (Fig. 1)^18,31^. A total of 79,080 compounds were tested at an average dose of 22.5 µM with the quality control parameter *Z*′-factor ranged from 0.55 to 0.84^32^ (Supplementary Tables 1 and 2). Among 29 other hits selected with efficacies >50% and potencies <20 µM, the activity of YC-001 showed a potency of 7.8 µM and an efficacy at 150–310% of the control activity score (Fig. 1e) that achieved a maximum within 15 h (Fig. 1f). Variability of YC-001’s efficacy of 150 to 310% was seen between experiments. No additive effect was seen with YC-001 and 9-*cis*-retinal co-treatment (Fig. 1g), suggesting a similar mechanism of action for these two compounds. Importantly, YC-001 activity was not affected when cells were exposed to light, whereas the activity of the 9-*cis*-retinal positive control required that cells be incubated in the dark.

### Confirmation of YC-001’s activity

**Fig. 2:**
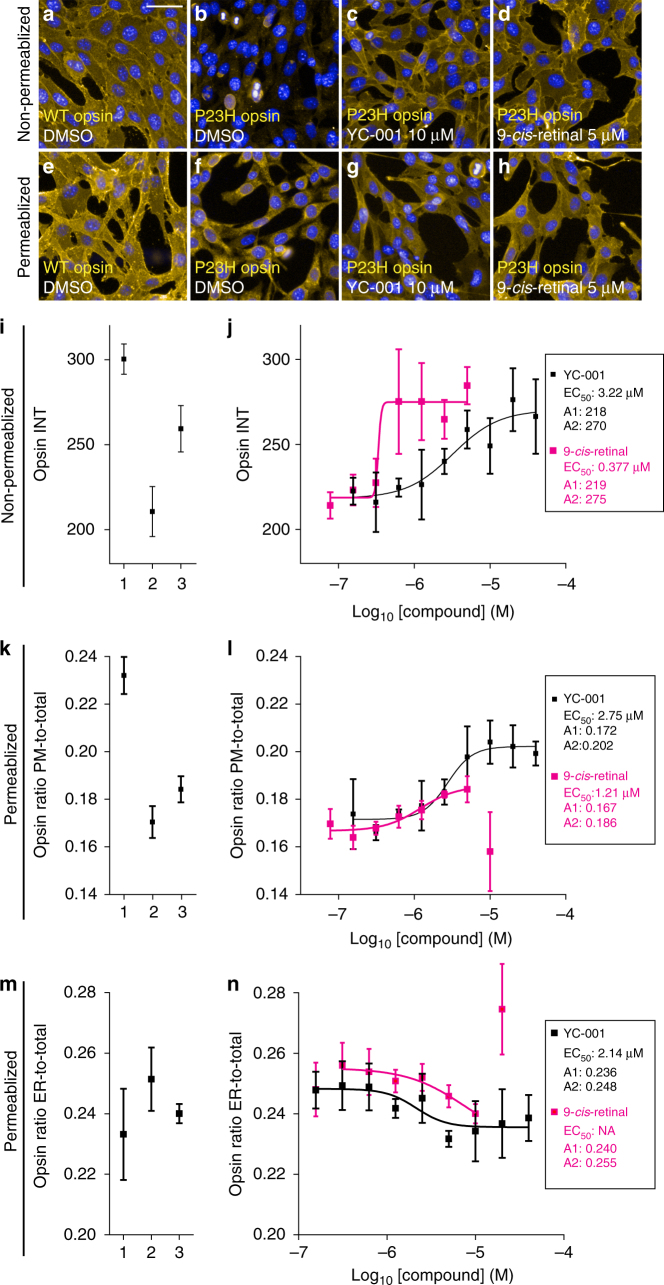
High-content imaging analysis of P23H-mutant opsin. **a**–**h** are fluorescence images of NIH3T3 cells expressing mouse WT or P23H-opsin imaged with Cy3 (yellow) and DAPI (blue). Scale bar, 50 μm. Images in **a**–**d** are from cells with rhodopsin immunostained on the cell surface only (non-permeabilized). Images in **e**–**h** were from cells with rhodopsin immunostained in the whole cell (permeabilized). Images **a**, **e** are from NIH3T3 cells expressing WT-opsin treated with 0.1% DMSO. Images **b**–**d** and **f**–**h** are from NIH3T3 cells expressing P23H opsin treated with 0.1% DMSO, 10 µM YC-001 or 5 µM 9-*cis*-retinal, left to right, respectively. Graphs (**i**–**n**). Graphs **i** and **j** were quantified from cell-surface immunostaining intensities of opsin on the plasma membrane (Opsin INT); graphs **k** and **l** are ratios of opsin staining on the plasma membrane compared to the whole cell from whole-cell immunostained images (Opsin ratio PM-to-total); graphs **m**, **n** are ratios of opsin staining in the ER region compared to whole-cell staining from whole-cell immunostained images (Opsin ratio ER-to-total). **i**, **k**, **m** Immunofluorescence intensities of opsin in controls: 1, NIH3T3 cells expressing WT-opsin treated with 0.1% DMSO; 2, NIH3T3 cells expressing P23H opsin treated with 0.1% DMSO; 3, NIH3T3 cells expressing P23H opsin treated with 5 µm 9-*cis*-retinal in the dark. Graphs **j**, **l**, **n** are quantifications of P23H opsin on the plasma membrane (**j**, **l**) or ER (**n**) of NIH3T3 cells, treated with a series of doses of YC-001 (black boxes) or 9-*cis*-retinal (magenta boxes). Values are averages of triplicate determinations, and error bars are s.d.s from those triplicates. Dose–response curves were fitted using Origin software with EC_50_ (μM), A1 (low plateau) and A2 (high plateau) of each compound listed in the inset box. This experiment was repeated twice

To confirm the activity of hit compounds identified by the HTS, high-content imaging analysis was used in NIH3T3 cells stably co-expressing mouse P23H opsin and green fluorescent protein (GFP) immunostaining the rod opsin mutant on the plasma membrane only, or in the whole cell. Images taken by both immunostaining methods showed that the P23H opsin on the plasma membrane was increased by treatment with either YC-001 or 9-*cis*-retinal in a dose-dependent manner (Fig. 2). Meanwhile, the ratio of P23H-opsin stain in the ER region to total P23H-opsin staining decreased by treatment with YC-001, suggesting that P23H opsin was mobilized from the ER to the plasma membrane, instead of just a change of its total amount (Fig. 2m, n). At a concentration higher than 10 µM, 9-*cis*-retinal euthanized most cells, whereas YC-001 up to 40 µM did not affect cell number. A total of 10 hit compounds were confirmed with activity rescuing the transport of P23H opsin (Supplementary Data 1) and none of these hit compounds resembled the chemical structure of 11-*cis*-retinal, the natural ligand and pharmacological chaperone of rod opsin. YC-001 (Fig. 1c), known as CID 2377702 in the PubChem database, was screened previously in 8 other bioassays but demonstrated no known activities (https://pubchem.ncbi.nlm.nih.gov). Owing to its novel pharmacological activity discovered in this study, YC-001 was selected for further investigation. Scriptaid, a pan-histone deacetylase inhibitor, also showed strong activity in rescuing P23H opsin transport (Supplementary Data 1). Moreover, co-treatment with YC-001 and scriptaid produced a synergistic effect, suggesting distinctive targets for these two active compounds^33^. YC-001 did not affect the amount or stability of the clarin-1 N48K mutant that causes Type III Usher syndrome^34^ in HEK 293 cells, suggesting the activity of YC-001 is specific to rod opsin (Supplementary Fig. 1).

### YC-001 improved the glycosylation of the P23H-opsin mutant

**Fig. 3:**
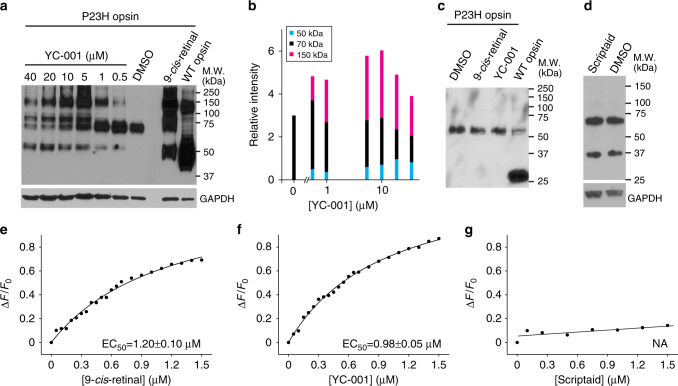
YC-001 improved the glycosylation profile of P23H opsin. **a** Effect of different treatments on immunoblots of lysates from NIH3T3 cells expressing WT or P23H opsin. Top panel, immunoblot of opsin; bottom panel, immunoblot of GAPDH. Lanes from left to right, immunoblots from a total of 15 µg lysate from NIH3T3 cells expressing P23H opsin that were treated with 40, 20, 10, 5, 1, or 0.5 µM YC-001, 0.1% DMSO, or 5 µM 9-*cis*-retinal, respectively; WT-opsin, immunoblot from a total of 5 µg lysate from NIH3T3 cells expressing WT-opsin treated with 0.1 % DMSO. **b** Relative intensities of P23H-opsin bands at 50 kDa (blue bars), 70 kDa (black bars) and 120 kDa (magenta bars) represented in cumulative bars as a function of YC-001 dosage. The band at 50 kDa is an opsin monomer with mature glycosylation; the band at 70 kDa is an opsin dimer with immature glycosylation; the band at 120 kDa is an opsin dimer with mature glycosylation. **c** Immunoblot of opsin from cell lysates deglycosylated by PNGaseF. Lanes from left to right, lysates from NIH3T3 cells expressing P23H opsin treated with either 0.1% DMSO, 5 µM 9-*cis*-retinal or 10 µM YC-001, respectively; WT-opsin, lysate from NIH3T3 cells expressing WT-opsin treated with 0.1% DMSO. **d** Immunoblot of P23H opsin from cells treated with 10 µM scriptaid or 0.1% DMSO, respectively. Immunoblot of GAPDH is shown on the bottom as a loading control. **e**–**g** Ligand-binding affects the chromophore-binding pocket of rod opsin. Bovine opsin within the ROS disc membranes was used for this assay. Trp fluorescence of opsin was measured both before and after addition of ligands (Supplementary Fig. 2). Changes of fluorescence intensity at 330 nm (Δ*F/F0*) are plotted as a function of the concentration of 9-*cis*-retinal (**e**), YC-001 (**f**), or scriptaid (**g**), respectively. Binding curves were fitted with the Hill function using Origin software. EC_50_s (μM) of each ligand were calculated and averaged from three biological repeats ± s.d.s and are indicated in the respective graphs. This experiment was repeated twice

Compromised transport of P23H opsin is associated with its lack of Golgi processing as a glycosylated transmembrane protein that is reflected by its shift in molecular mass differing from that of WT-opsin as detected by immunoblotting (Fig. 3a, lanes 7 and 9)^18,19,35^. Upon treatment with increasing concentrations of YC-001, three bands at 50, 75, and 120 kDa emerged in immunoblots of cell lysates expressing P23H opsin (Fig. 3a, lanes 1–6). Although the bands at 50 and 120 kDa were also detected in immunoblots of WT-opsin and P23H opsin from cells treated with 9-*cis*-retinal, consistent with mature glycosylated monomer and dimer forms of opsin, the band at 75 kDa could represent an immature intermediate product of glycosylated P23H-opsin dimer formed upon treatment with YC-001. Quantitatively, the total amount of P23H opsin reached a maximum when treated with 5 or 10 µM YC-001 (Fig. 3b). PNGaseF incubations confirmed that shifted molecular mass of P23H opsin by treatment of YC-001 or 9-*cis*-retinal was due to the improvement of glycosylation (Fig. 3c). In contrast, treatment with scriptaid does not affect the glycosylation of this mutant opsin (Fig. 3d). The glycosylation of opsin varies between cell types in which it is expressed^13,19,33^. Here we used NIH3T3 cells to show that immature glycosylated P23H rod opsin can be further processed in cells treated with 9-*cis*-retinal or YC-001.

### YC-001 reversibly binds rod opsin

To test whether YC-001 binds rod opsin and act as a pharmacological chaperone, a ligand-binding assay was performed using Trp fluorescence to monitor the chromophore pocket conformation in bovine opsin^36^. Titration of opsin in the disc membranes with either 9-*cis*-retinal or YC-001 quenched the Trp fluorescence at 330 nm corresponding to the conformational change of Trp 265 in the chromophore pocket (Fig. 3e, f and Supplementary Fig. 2) The *EC*_*50*_ of YC-001 was 0.98 ± 0.05 µM (Fig. 3f), comparable to the *EC*_*50*_ of 9-*cis*-retinal to rod opsin at 1.20 ± 0.10 µM (Fig. 3e). This finding suggested that YC-001 either binds to opsin in the chromophore pocket, or its allosteric binding affects the chromophore pocket conformation. In contrast, scriptaid did not affect Trp fluorescence, suggesting that it does not bind to rod opsin (Fig. 3g; Supplementary Fig. 2c).

**Fig. 4:**
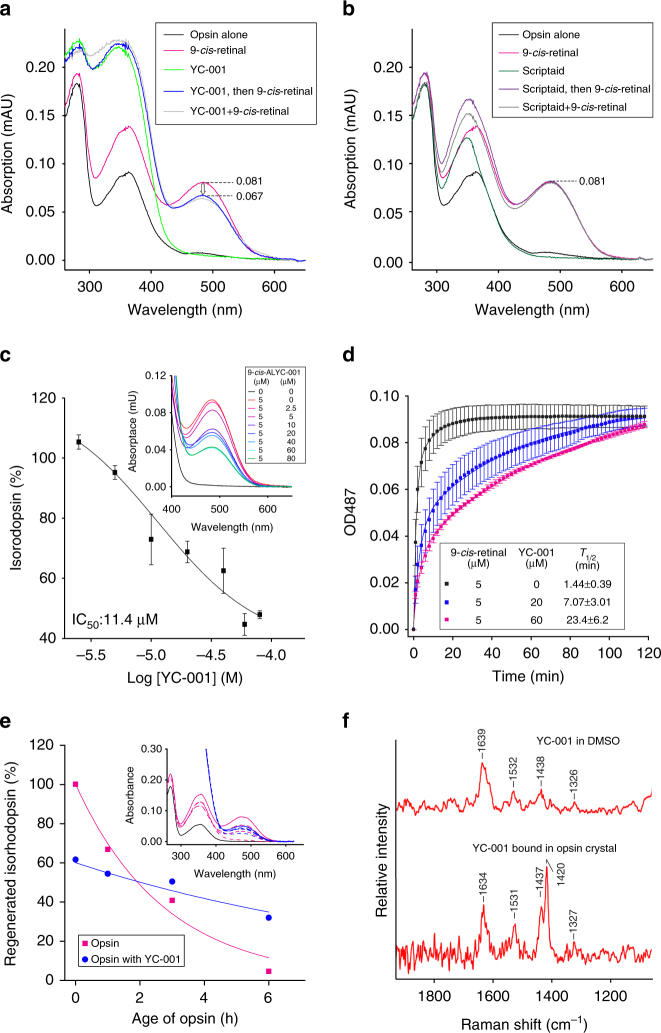
YC-001 delays isorhodopsin pigment regeneration. Bovine opsin (2.5 µM) in ROS membranes was incubated with compounds (20 µM) for 30 min at RT. After membrane solubilization, absorbance at 487 nm was recorded to measure the amount of isorhodopsin. **a** UV–visible absorption spectra of opsin (black) and opsin treated with 9-*cis*-retinal (magenta), YC-001 (light green), YC-001 followed by 9-*cis*-retinal for 15 min each (blue), and a mixture of YC-001 and 9-*cis*-retinal (gray). **b** UV–visible absorption spectra of opsin treated with 9-*cis*-retinal (magenta), scriptaid (dark green), scriptaid followed by 9-*cis*-retinal for 15 min each (purple), and a mixture of 9-*cis*-retinal and scriptaid (gray). **c** Percentage of regenerated isorhodopsin from sequential treatment with YC-001 and 9-*cis*-retinal for 15 min each as a function of YC-001 concentration in a log format. Isorhodopsin regenerated with 9-*cis*-retinal alone was normalized as 100%. Values and error bars were averages and s.d.s from three biological replicates. Inset, absorption spectra of opsin with 5 μM 9-*cis*-retinal and 0 (red), 2.5 (pink), 5 (magenta), 10 (purple), 20 (dark blue), 40 (cyan), 60 (light blue), or 80 μM YC-001 (green), respectively. **d** Time course of isorhodopsin regeneration in the presence of 0, 20, or 60 μM YC-001 followed by addition 5 μM 9-*cis*-retinal for 15 min each (black, blue, and magenta boxes, respectively). Values and error bars were averages and s.d.s of three biological repeats. Data were fitted with second-order exponential decay and apparent half-lives (*T*_1/2_ ± standard error) are shown in the inset box. **e** Percentage of regenerated isorhodopsin from aged opsin (magenta) or opsin incubated with YC-001 (blue) at RT for 0, 1, 3, and 6 h before regeneration with 9-*cis*-retinal. Isorhodopsin regenerated from opsin at 0 h was set at 100%. Plots of regenerated isorhodopsin levels were fitted by the exponential decay function. The inset shows the absorption spectra of regenerated isorhodopsin from aged opsins. Black, opsin alone. **f** Raman spectrum of YC-001 in DMSO solution (top) and a difference spectrum after subtracting the spectrum of rod opsin crystal from that of opsin crystal soaked with YC-001 (bottom). Each experiment was repeated twice

To test if YC-001 is competitive with 9-*cis*-retinal for binding to rod opsin, pigment regeneration of opsin was monitored in ROS disc membranes by absorption spectroscopy. The peak at 487 nm was due to Schiff base linkage formation between 9-*cis*-retinal and the K296 in the chromophore pocket, which was used to quantify the regenerated isorhodopsin (Fig. 4a, b)^25,37^. An absorption at 340 nm was seen when YC-001 was dissolved in the buffer or added to rod opsin in disc membranes (Fig. 4a, light green line), suggesting that YC-001 does not form a chromophore analog when bound to opsin. The absorption at 487 nm was reduced when an increasing concentration of YC-001 was added with 5 μM 9-*cis*-retinal simultaneously or sequentially to rod opsin in disc membranes (Fig. 4a, blue and gray lines and Fig. 4c), suggesting that less isorhodopsin was generated in the presence of YC-001 competing with 9-*cis*-retinal in a short timeframe. When scriptaid and 9-*cis*-retinal were added simultaneously or sequentially to rod opsin in disc membranes, the absorption peak at 487 nm overlapped with that regenerated with only 9-*cis*-retinal (Fig. 4b), indicating that scriptaid does not compete with 9-*cis*-retinal in the chromophore-binding pocket.

The kinetics of isorhodopsin regeneration was traced when disc membranes were treated with YC-001 and 9-*cis*-retinal sequentially (Fig. 4d). An increasing concentration of YC-001 treatment did not affect the total amount of regenerated isorhodopsin pigment at the end the reaction, but slowed down the kinetics of pigment regeneration, as demonstrated by an increase in apparent half-life.

Disc membranes were then treated either with YC-001 and 9-*cis*-retinal individually, simultaneously or sequentially for a total of 30 min, followed by opsin or isorhodopsin purification. The purified isorhodopsin by treatment with YC-001 and 9-*cis*-retinal showed a prominent absorption at 487 nm with a small shoulder at 340 nm, suggesting that most of the YC-001 was washed out during opsin/isorhodopsin purification (Supplementary Fig. 3). This result confirmed that the interaction between YC-001 and opsin is reversible.

Whereas rhodopsin is quite stable, rod opsin is unstable at room temperature (RT). While aging at RT, YC-001 treated rod opsin showed a significantly longer half-life compared to that of opsin alone (Fig. 4e), suggesting YC-001 stabilizes the rod opsin structure.

To further characterize the molecular binding of YC-001 to rod opsin, we attempted to obtain the YC-001:opsin complex as a crystal structure but with little success. Using Raman spectroscopy, vibrational modes from YC-001 were clearly seen in the difference spectrum obtained by subtracting the rod opsin spectrum from the complex spectrum, in comparison to the spectrum of YC-001 in DMSO (Fig. 4f). Vibrational modes of YC-001 did not shift significantly when detected in the YC-001:opsin crystal, but these peaks were narrower than those in the free YC-001 spectrum, suggesting the non-covalent binding of YC-001 to rod opsin^38,39^.

### YC-001 is an inverse agonist and antagonist of rod opsin

**Fig. 5:**
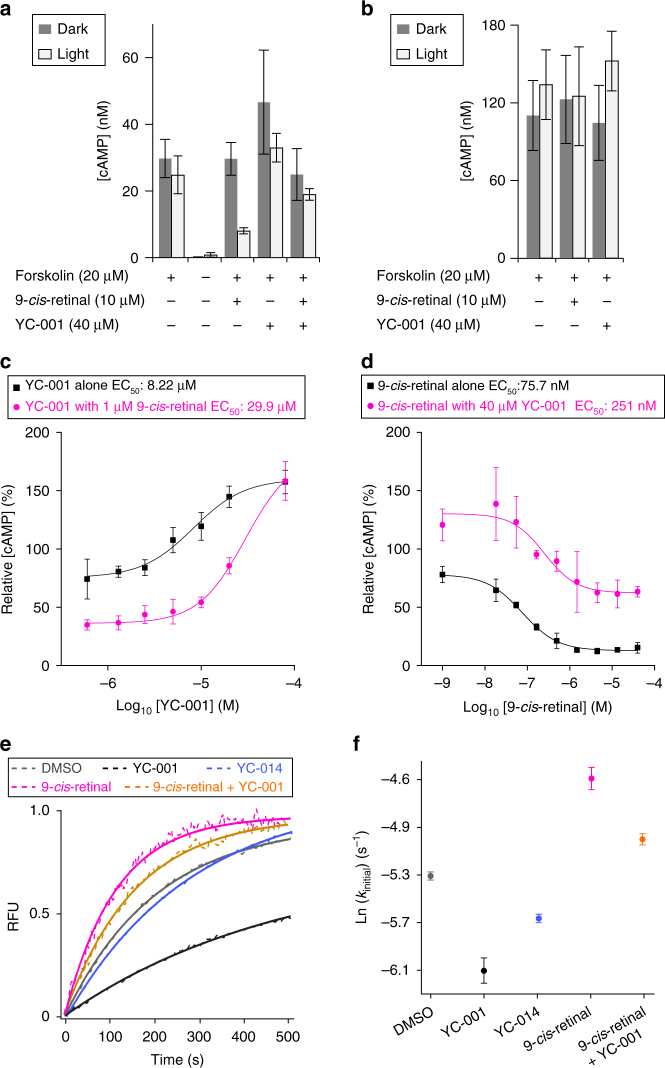
YC-001 is an inverse agonist and antagonist to rod opsin. Rhodopsin couples to *G*_*i*/*o*_ signaling in a light-dependent manner leading to the reduction of cAMP level in mammalian cells. Forskolin was added to the cells to saturate their cAMP levels. **a** Levels of cAMP in NIH3T3-(Opsin/GFP) cells treated as noted under the chart. Cells treated in the dark and in light were in gray and white bars, respectively. Bar values are the averages of three replicates, and error bars are s.d.s of the replicates. **b** Levels of cAMP in NIH3T3-(GFP) cells treated with PBS, 10 µM 9-*cis*-retinal, or 40 µM YC-001, respectively. **c** cAMP levels in NIH3T3-(Opsin/GFP) cells treated with a series of YC-001 doses in the presence (magenta circles) or absence of 1 µM of 9-*cis*-retinal (black squares) under light. Doses of YC-001 tested were 80, 20, 10, 5, 2.5, 1.25, 0.625, and 0.313 µM. The cAMP level in cells treated with forskolin only was normalized as 100%, and that treated without forskolin as 0%. **d** cAMP levels in NIH3T3-(Opsin/GFP) cells treated with a dose series of 9-*cis*-retinal in the presence (magenta circles) or absence of 40 µM of YC-001 (black squares) under light. Doses of 9-*cis*-retinal tested were 40, 13.3, 4.44, 1.48, 0.494, 0.165, 0.055, 0.018, and 0.001 µM. **e** *G*_*t*_ activation by bovine rod opsin or isorhodopsin. Constitutive activity of bovine opsin in disc membranes or photoactivated isorhodopsin activity was recorded by fluorescence with excitation and emission at 300 and 345 nm, respectively, as a function of time, due to GTPγS-induced dissociation of the opsin/isorhodopsin: *G*_*t*_ complex. Dashed experimental lines were fitted by the first-order exponential decay functions shown in solid lines. Each condition was repeated in three biological replicates and initial rates and error bars were averages and s.d.s. shown in **f**. Opsin were treated with DMSO (gray), 40 µM YC-001 (black), 40 µM YC-014 (blue), 40 µM 9-*cis*-retinal (magenta), and a mixture of 40 µM 9-*cis*-retinal and 40 µM YC-001 (orange). Each experiment was repeated twice

While acting as a pharmacological chaperone, does YC-001 binding also affect intracellular signaling of rod opsin? Here, a cAMP assay was used to address this issue (Fig. 5). In mammalian cells, heterologously expressed isorhodopsin couples to the endogenous Gi/o signaling cascade when activated by light^19,38^. The NIH3T3-(WT-opsin/GFP) cells (Fig. 5a) exhibited lower cAMP level than the NIH3T3-(GFP) cells (Fig. 5b), suggesting the basal activity of rod opsin through the activated Gi/o pathway that inhibits adenylate cyclase, responsible for the synthesis of cAMP. Upon light exposure, the NIH3T3-(WT-opsin/GFP) cells treated with 9-*cis*-retinal showed significantly reduced cAMP level, confirming that isorhodopsin had activated Gi/o signaling (Fig. 5a). However, YC-001 treated NIH3T3-(WT-opsin/GFP) cells evidenced a dose-dependent increase in cAMP levels as compared to non-treated cells (Fig. 5b, c) with an EC_50_ value of 8.22 µM, either in the dark or light, suggesting that YC-001 silences the basal activity of rod opsin. Co-treated with 1 µM 9-*cis*-retinal under light, YC-001 also induced a dose-dependent elevation in cAMP levels in NIH3T3-(WT-opsin/GFP) cells under light exposure (Fig. 5c), suggesting that YC-001 antagonizes the isorhodopsin formation or the photoactivation capabilities of isorhodopsin. The 9-*cis*-retinal dose–response curve with 40 μM YC-001 co-treatment revealed a 3-fold increase in the EC_50_, and the curve shifted upward compared to the dose curve obtained by 9-*cis*-retinal treatment alone (Fig. 5d). Together, to our knowledge, YC-001 is the first non-retinal compound that has been revealed to have both inverse agonist and antagonist activity toward rod opsin.

To confirm the inverse agonist and antagonist activity of YC-001 toward rod opsin, the initial rate of *G*_*t*_ activation was measured by a fluorescence change due to GTPγS uptake leading to dissociation of the *G*_*t*_:opsin complex^39,40^. Bovine rod opsin has a basal activity for *G*_*t*_ activation^41^. Upon treatment with 40 µM YC-001, the initial rate of *G*_*t*_ activation for opsin was substantially reduced (Fig. 5e, f, black line and point, ln(*k*_initial_) *=* −6.1) compared to the DMSO control group (Fig. 5e, f, gray line and point, ln(*k*_initial_) *=* −5.3), confirming that YC-001 silenced the constitutive activity of rod opsin. Moreover, opsin treated with YC-014 lacking pharmacological chaperone activity (Supplementary Data 2) at 40 µM also showed a slightly decreased rate of *G*_*t*_ activation (Fig. 5e, f, blue line and point, ln(*k*_initial_) = −5.7), which could be due to weak binding of YC-014 to rod opsin that is not sufficient to stabilize the P23H-opsin mutant. When co-treated with 40 µM 9-*cis*-retinal and 40 µM YC-001, regenerated isorhodopsin showed a reduced rate of *G*_*t*_ activation upon illumination (Fig. 5e, f, orange line and point, ln(*k*_initial_) = −4.9) compared to that regenerated by 9-*cis*-retinal alone (Fig. 5e, f, magenta line and point, ln(*k*_initial_) = −4.6), confirming that YC-001 antagonizes isorhodopsin coupled *G*_*t*_ activation.

### YC-001 protects *Abca4*^*−/−*^*Rdh8*^*−/−*^ mice from retinal damage

**Fig. 6:**
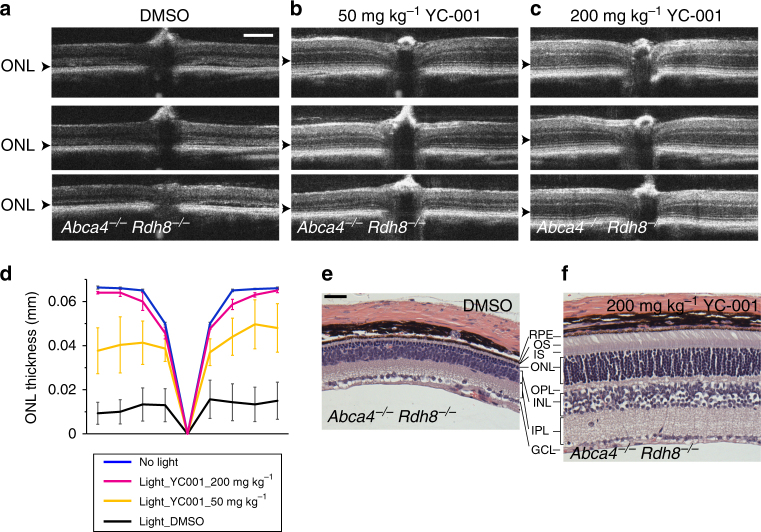
YC-001 protects *Abca4*^*−/−*^*Rdh8*^*−/−*^ mouse retinas from light damage. Owing to the loss of both ABCA4 and RDH8, all-*trans*-retinal cannot be efficiently cleared from the ROS of *Abca4*^*−/−*^*Rdh8*^*−/−*^ mice. Thus, their retinas undergo degeneration upon exposure to intense light. Here, *Abca4*^*−/−*^*Rdh8*^*−/−*^ mice were treated with either DMSO or YC-001 i.p. 30 min before exposure to 10,000 lux light for 30 min. SD-OCT images were taken seven days after light exposure (**a**–**d**). Mice then were euthanized and their eyes were used for histological examination (**e**, **f**). **a** SD-OCT images from mice treated with 50 µL DMSO. Arrowheads indicate significantly degenerated ONL. Scale bar, 200 μm. **b**, **c** SD-OCT images from mice treated with 50 or 200 mg kg^−1^ bw of YC-001, respectively. **d** Plots of ONL thickness from SD-OCT images in (**a**–**c**). Lines represent averaged ONL thicknesses from three mice and error bars are the s.d.s. *n* = 3. **e** HE staining of *Abca4*^*−/−*^*Rdh8*^*−/−*^ mouse retina seven days after pre-incubation in DMSO and exposure to 10,000 lux light. Scale bar, 100 μm. **f** HE staining of *Abca4*^*−/−*^*Rdh8*^*−/−*^ mouse retina seven days after pretreatment with 200 mg kg^−1^ bw YC-001 and exposure to 10,000 lux light. RPE retinal pigmented epithelium, OS outer segment, IS inner segment, ONL outer nuclear layer, OPL outer plexiform layer, INL inner nuclear layer, IPL inner plexiform layer, GCL ganglion cell layer. This experiment was repeated twice

Owing to the abundance and physiological significance of rhodopsin in ROS, its homeostasis is closely connected with photoreceptor survival. Thus, YC-001 as a pharmacological chaperone and modulator of rod opsin should also protect photoreceptors by stabilizing bleached opsin and antagonizing phototransduction activity in light-induced models of retinal degeneration. We previously developed a bright light-induced retinal degeneration model for pharmacological testing^42–44^. Here, 6-week-old *Abca4*^*−/−*^*Rdh8*^*−/−*^ mice, a model characterized by its increased susceptibility to bright light-induced photoreceptor degeneration, were preconditioned with YC-001 at two doses: 50 or 200 mg kg^−1^ body weight (bw) by intraperitoneal (i.p.) injection along with DMSO as a vehicle control. Thirty min after treatment, mice were exposed to bright light (10,000 lux) for 30 min. Seven days later, retinal structures of these mice were imaged by spectral domain-optical coherence tomography (SD-OCT) (Fig. 6a–d) and histological hematoxylin and eosin (HE) staining (Fig. 6e, f). Although DMSO-treated mouse retinas featured significantly diminished outer nuclear layers (ONLs) (Fig. 6a, d, e), indicating the loss of photoreceptor cells, YC-001-treated mice evidenced a dose-dependent protection of the ONL from light-induced damage (Fig. 6b–d, f). These findings demonstrate that YC-001 protects *Abca4*^*−/−*^*Rdh8*^*−/−*^ mice from bright light-induced retinal degeneration.

### YC-001 is detected in the mouse retina

**Fig. 7:**
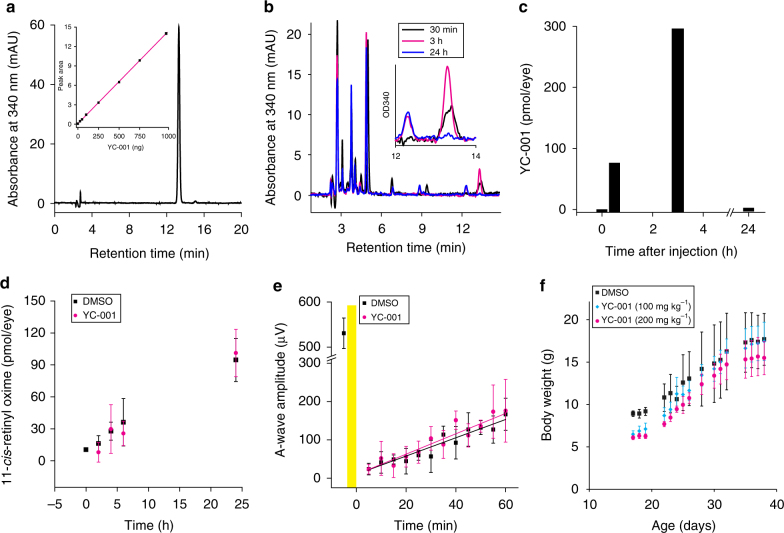
YC-001 enters mouse eyes without affecting the visual cycle. **a** HPLC chromatogram of a YC-001 standard indicating a peak at a retention time of 13.2 min with an absorbance at 340 nm. Inset shows the standard curve of YC-001 hexane extracts with its peak area versus its weight in ng. **b** HPLC chromatogram of hexane extracts from four six-week-old C57BL/6 mouse eyes 0.5, 3, or 24 h after i.p. injection with YC-001 at 200 mg kg^−1^ bw in black, magenta, and blue, respectively. The inset is an enlarged chromatogram of the peaks with retention times from 12 to 14 min. **c** Amounts of YC-001 in pmol per eye plotted as a function of time after injection with YC-001. Time 0 denotes mice not injected with YC-001. **d** Amounts of 11-*cis*-retinyl-oxime representing the relative amounts of regenerated rhodopsin pigment were plotted as a function of time after bleaching. Six-week-old C57BL/6 mice were injected with 200 mg kg^−1^ bw YC-001 (magenta) or 50 µL DMSO i.p. 30 min before their exposure to 10,000 lux light for 10 min. Mice then were placed in the dark and euthanized at 0, 2, 4, 6, and 24 h after bleaching. Retinyl-oximes were extracted from homogenized eyes and separated by HPLC. **e** Recovery of mouse scotopic ERG a-wave amplitude plotted as a function of time after bleaching. Dark-adapted C57BL/6 mice received YC-001 (200 mg kg^−1^ bw) or DMSO by ip injection 1 h before light exposure. Mice with dilated pupils were then exposed to 2,000 lux light for 5 min. Yellow shade represents 5 min illumination. Scotopic a-wave amplitude from unbleached dark-adapted mice was shown before time 0. **f** Bw of YC-001 or DMSO-treated mice plotted as a function of their age. C57BL/6 mice were treated with 100 or 200 mg kg^−1^ bw YC-001 by daily i.p. injections, starting on Day 14. Black, DMSO; blue, 100 mg kg^−1^ YC-001; magenta, 200 mg kg^−1^ YC-001. Values and error bars were from averages and s.d.s, *n* = 3. Each experiment was repeated twice

A high-performance liquid chromatography (HPLC) (Fig. 7a–c) and mass spectrometry (MS) (Supplementary Fig. 4) analysis was performed to determine if YC-001 can be detected in C57BL/6 mouse eyes after systemic administration. About 70 pmol per eye of YC-001 was detected at 0.5 h after i.p. injection at 200 mg kg^−1^ bw, increasing to 280 pmol per eye at 3 h, and then diminishing to an undetectable level by 24 h (Fig. 7c). This result confirmed that YC-001 enters mouse eyes after systemic administration but is not retained for prolonged periods.

### YC-001 does not inhibit the visual or retinoid cycle

To test whether YC-001 affects the visual cycle, 11-*cis*-retinoid regeneration was analyzed from photobleached mice treated with YC-001 (Fig. 7d). Compared to the DMSO control group, the YC-001-treated group showed little difference in the recovery of 11-*cis*-retinyl-oxime, suggesting that YC-001 does not affect the visual chromophore regeneration.

### YC-001 does not delay rhodopsin regeneration in vivo

To determine whether YC-001 antagonizes rhodopsin signaling in vivo, the scotopic electroretinogram (ERG) recovery after bleaching was recorded in mice treated with either YC-001 or DMSO. Compared to a non-bleached age-matched control group, both YC-001- and DMSO-treated mice showed significant reductions of the initial scotopic ERG responses (Fig. 7e) followed by a linear increase of the a-wave responses over 1 h with similar recovery rates. Considering that scotopic ERG a-wave responses directly represent a rod photoreceptors’ response to light, YC-001 did not inhibit rhodopsin regeneration after bleach.

### YC-001 shows no acute toxicity

To test whether high doses of YC-001 could cause severe toxicity, we administered YC-001 (200 and 100 mg kg^−1^ bw) or DMSO to C57BL/6 mice by daily i.p. injections from day 14 to 38 after birth. All mice survived the treatment period with no obvious behavioral or bw growth changes when comparing the YC-001-treated groups and DMSO group (Fig. 7f), suggesting virtually no acute toxicity of YC-001.

### YC-001 shows no evidence of mutagenicity

To assess the risk of tumorgenesis by YC-001 treatment, Ames bacterial mutation tests were performed in a total of five bacteria strains^45^. The negative results of YC-001 in the Ames test (Supplementary Table 3) suggests that YC-001 has a low risk of mutagenicity if tested in vivo.

### YC-001 does not affect cyclooxygenase 1 activity

The furanone ring of YC-001 is also seen in the nonsteroidal anti-inflammatory drugs inhibiting cyclooxygenase (COX) enzymes^46^, thus one can argue the retinal protection of YC-001 may be due to its anti-inflammatory effects via inhibition of COX-1 that is expressed in all tissues. We measured and did not see changes of the COX-1 enzymatic activity in the presence of up to 80 µM of YC-001 (Supplementary Fig. 5).

### YC-001 showed clearance by phase I hepatic enzymes

Even though EC_50_ of YC-001 is at the micromolar level in vitro, its efficacy in the *Abca4*^*−/−*^*Rdh8*^*−/−*^ mice required a higher dose of systemic administration. To address the difference of effective dosage of YC-001 in vitro and in vivo, we characterized the pharmacokinetics of YC-001 in C57BL/6 mice by i.p. injections. A first-order elimination of YC-001 was observed in the mouse plasma featured with a short half-life (*T*_1/2_) at 34.5 min and an initial plasma concentration (*C*_0_) at 7.28 µg mL^−1^. Using the *T*_1/2_ and *C*_0_, we further estimated the elimination rate constant (*K*_e_), the volume of distribution (*V*_d_) and clearance. The high clearance of YC-001 at 0.552 L min^−1^ kg^−1^ bw was due to a high *K*_e_ at 0.0201 min^−1^ and a large *V*_d_ at 27.5 L kg^−1^ bw (Supplementary Fig. 6). Although the large *V*_d_ is due to the hydrophobicity of YC-001, the high elimination rate constant suggests a high rate of metabolism or secretion by the liver and kidney. Indeed, by measuring the stability of this compound in isolated mouse and human liver microsomes, we found that YC-001 has an even higher initial clearance compared to the fast clearance drug, verapamil (Supplementary Fig. 7). The main metabolite of YC-001 showed an increased molecular mass by 16, suggesting the addition of an oxygen atom to YC-001.

### YC-001 rescues multiple rod opsin mutants in adRP

**Fig. 8:**
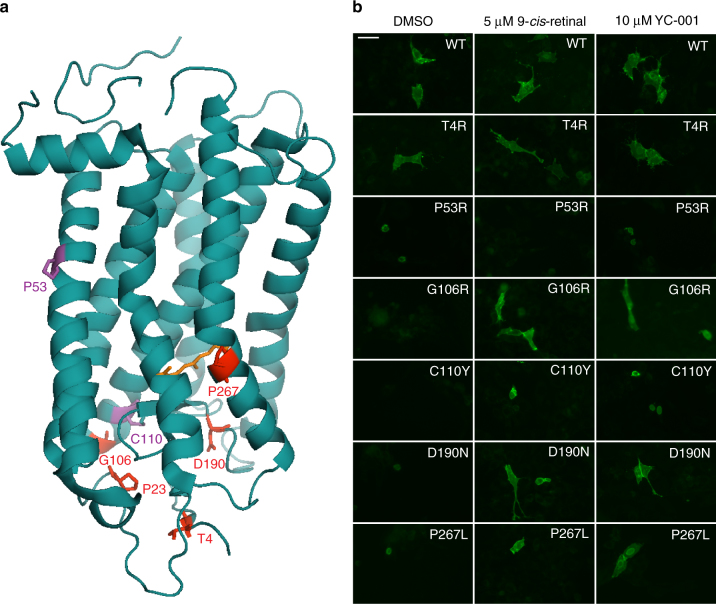
Effect of YC-001 on the transport of rod opsin mutants. **a** Illustration of seven autosomal dominant retinitis pigmentosa associated mutation sites on the bovine rhodopsin crystal structure (PDB ID: 1f88). The overall structure of rhodopsin is shown in blue with 11-*cis*-retinal labeled in orange. Side chains of T4, P23, G106, D190, and P267 are labeled in red, and side chains of P53 and C110 are labeled in magenta. **b** Cell-surface immunostained images of rod opsin mutants expressed in NIH3T3 cells exposed to DMSO, 9-*cis*-retinal or YC-001. Cells transfected with human rhodopsin WT or mutants were treated with DMSO (0.1%) or YC-001 (10 µM) for 24 h. Cells were fixed and only rod opsin on the cell surface was immunostained with Alexa 488-conjugated B6-30 anti-rhodopsin antibody. Green fluorescence images were taken under a ×20 objective. 9-*cis*-retinal was tested at 5 μM and YC-001 at 10 μM, as labeled in each panel. This experiment was repeated twice

To test whether YC-001 rescues other rhodopsin misfolding mutations than the P23H, a total of six Class II mutations of human rod opsin were generated: T4R, P53R, G106R, C110Y, D190N, and P267L (Fig. 8a). NIH3T3 cells were transfected with WT or mutant opsin constructs followed by treatment with DMSO or YC-001. Cell-surface immunostaining of rod opsin was imaged by fluorescence microscopy. Upon DMSO treatment, all rod opsin mutants except T4R showed dim background fluorescence, whereas immunostaining of WT-opsin and T4R opsin were clearly seen on the cell membrane (Fig. 8b). Under YC-001 treatment, cell-surface staining of G106R, D190N, and P267L, but not P53R or C110Y mutants increased significantly (Fig. 8b). Differences in YC-001’s efficacy between each mutant could reflect variations between their folding defects. Residues P23, G106, and D190 are located on the extracellular/intradiscal side of the rhodopsin structure^25^ surrounding the anti-parallel β-plug of the retinal binding pocket, and YC-001 was able to rescue the transport in these mutants. Residues P53, and P267 are located on the transmembrane helixes, but only P267L with its sidechain facing towards the chromophore-binding pocket was rescued by YC-001. Residue C110 forms the only disulfide bond of rhodopsin with C187, which is essential to stabilize the entire structure of the protein, and YC-001 was not effective for this mutant. Varying efficacies of YC-001 among these six class II mutants suggest that the structural stability defects among class II mutations differ and may require different small-molecule chaperones for their stabilization.

### Medicinal chemistry of YC-001

To improve its efficacy, potency, and solubility, a medicinal chemistry study of YC-001-related compounds was undertaken. YC-001 analogs obtained from commercial vendors or by customized synthesis were denoted as YC-002 to YC-063. YC-001 has three chemical moieties (Fig. 1c): I, a 2-chlorothiophene ring; II, a furan-2(5H)-one ring; and III, a thiophene ring. Each of these moieties was modified individually. Activities of YC compounds in rescuing P23H-opsin transport were then tested with the β-Gal fragment complementation assay (Supplementary Data 2–5). Changes in moiety III were better tolerated and retained the activity as pharmacological chaperones of opsin (Supplementary Data 2), whereas moieties II and I were more resistant to changes (Supplementary Data 3 and 4), except for some activity that remained in YC-002, YC-021, and YC-049 (Supplementary Data 3). Notably, among compounds modified in moiety III, activity was preserved or improved by substitutions with 3-thiophene (YC-051), a phenyl ring (YC-047), or 2-pyridine (YC-043), whereas substitution with furan (YC-028) failed to retain activity. Among phenyl substitutions derived from YC-047, *ortho*-modifications on the phenyl ring retained relatively higher efficacies (YC-068, YC-032, YC-013, and YC-033), whereas the corresponding meta-modifications displayed lower efficacies (YC-050 and YC-034), and *para*-modifications had little efficacy (YC-053). Substitutions on the phenyl ring of YC-047 favored the sequence –F = –Cl > –NO_2_ > –OMe, if modified at the same position. Interestingly, the pyridine substitutions of moiety III also revealed that activity was preserved with 2-pyridine (YC-047) but not 3- or 4-pyridine (YC-030 or YC-031). In summary, we hypothesize that moieties II and I of YC-001 bind in a small region of the opsin pocket, allowing only minor changes, whereas moiety III resides in a relatively larger part of the binding pocket, thereby tolerating a larger spectrum of modifications. The S atom in moiety III might interact with opsin. The medicinal chemistry study of YC-001 also yielded four active compounds with improved potency, efficacy, or solubility (YC-043, YC-068, YC-032, and YC-054). These compounds might be especially useful for future crystallography and in vivo studies.

## Discussion

Rhodopsin pigments, densely packed in an organized array in ROS disc membranes, are renewed in the retina over a 10-day cycle^47,48^. Any reduction in the biosynthesis of rhodopsin leads to shortened ROS^49^, clearly indicating that rhodopsin homeostasis is closely coupled with ROS morphology. In the case of adRP associated with the rhodopsin P23H mutation, photoreceptor cell death occurs over a prolonged period and can result from either of two forms of cell stress: (1) ER stress in response to continuous proteolysis of the structurally unstable opsin mutant^9,12,50^; and (2) cellular stress due to disrupted ROS disc membrane organization associated with the aggregation of P23H opsin following photo- or thermal-bleaching^13,19,51^. Pharmacological chaperones can mitigate the effects of misfolded proteins and restore function. An example is the recent discovery of a novel small-molecule stabilizer of clarin-1 that can rescue hearing in a mouse model carrying the clarin-1 mutation N48K commonly found in type III Usher syndrome^34^. Extensive efforts have been made to discover pharmacological stabilizers of rhodopsin for the treatment of RP. A vitamin A supplementation trial in 1993 showed beneficial effects for RP patients^52,53^, suggesting that a sufficient supply of 11-*cis*-retinal can delay retinal degeneration. Ligand-based development and virtual screening have yielded analogs of 11-*cis*-retinal, most retaining the aldehyde tail for Schiff base linkage with rod opsin^54–56^. The limitation of 11-*cis*-retinal supplementation is that the rescue of rhodopsin folding requires the absence of light, because the chromophore is hydrolyzed from rod opsin after a bleach. Therefore, a non-retinoid pharmacological chaperone would help improve the stability of rod opsin, both during its biosynthesis in the ER and after it’s bleached in ROS.

Here, we report the discovery of YC-001, a non-retinoid ligand of rod opsin that rescued the transport and glycosylation of the P23H rod opsin mutant in mammalian cells. Significantly, YC-001 binds rod opsin non-covalently without regulation by light. Because regeneration of rhodopsin involves covalent bond formation, whereas YC-001 binds to opsin non-covalently, the total amount of regenerated rhodopsin will not be affected by YC-001 treatment if given sufficient time. Therefore, this observation can explain why there is no additive effect when YC-001 was co-treated with 9-*cis*-retinal (Fig. 1g). In this manner, the pharmacological chaperone activity of YC-001 could stabilize the P23H opsin both during its biosynthesis and after the mutant opsin has been bleached in the ROS discs. In contrast, for the other active compound scriptaid that also increases the transport of P23H opsin to the plasma membrane, there is no evidence to show that it binds to rod opsin. First, scriptaid does not affect the fluorescence of Trp 265 in rod opsin; second, scriptaid does not compete with 9-*cis*-retinal for isorhodopsin regeneration; third, scriptaid does not affect the glycosylation of P23H opsin; and finally, the synergy between scriptaid and YC-001 on the transport of P23H opsin suggests different mechanisms of actions between the two molecules^33^. In our previous report, scriptaid as a histone deacetylase inhibitor, affected a total of 6% of the entire transcriptome^33^. Rather than directly binding to rod opsin, scriptaid may improve P23H opsin transport through transcriptional regulation of multiple pathways including cytoskeleton dynamics, proteolysis, and vesicle transport.

To our knowledge, YC-001 is the first non-retinoid modulator of rod opsin activity that can serve as a novel pharmacological tool to study the role of rhodopsin signaling in normal and diseased conditions. For example, slowing the visual cycle has shown therapeutic potential for Stargardt disease, which lacks a functional all-*trans*-retinal flippase, ABCA4^28,29,57,58^. Different from previously developed modulators of retinoid cycle components, YC-001 could serve as a novel drug candidate by slowing the regeneration of rhodopsin and thus reducing all-*trans*-retinal production stimulated by light. We found that just a single dose of YC-001 protected the retinas of *Abca4*^*−/−*^*Rdh8*^*−/−*^ mice from bright light-induced retinal degeneration. Lacking ABCA4 and retinol dehydrogenase 8 (RDH8), these double knockout mice have defects in clearing all-*trans*-retinal released from photobleached rhodopsin, thereby making photoreceptors vulnerable to bright light damage due to all-*trans-*retinal’s acute cytotoxicity^44^. The protection observed with YC-001 is likely due to its antagonistic activity that competes with 11-*cis*-retinal for pigment regeneration and phototransduction. Because YC-001 binds to rod opsin reversibly, whereas 11-*cis*-retinal binds and forms a Schiff base linkage with rod opsin, competition between the two molecules should ultimately favor the latter. Therefore, we observed competition between YC-001 and 9-*cis*-retinal for opsin binding in vitro (over a 15 to 30 min period), but not a reduction of 11-*cis*-retinyl-oxime released from dark-adapted bleached retinas treated with YC-001 relative to the DMSO control group (timeframe in hours), or any scotopic ERG changes manifested upon YC-001 treatment in vivo. The retinal protection of *Abca4*^*−/−*^*Rdh8*^*−/−*^ mice suggests a therapeutic potential for YC-001 in light-induced retinal degenerations.

*P23H/*^*+*^ knock-in mice serve as an adRP model associated with the human P23H rhodopsin mutation. Degeneration of rod photoreceptors in these mice takes about 4 to 5 months^13^, resulting in a prominent loss of the ONL and visual function. This progressive degeneration provides a therapeutic timeframe from 2 weeks to 4 months of age. The main challenge here is to develop a treatment that will maintain an efficacious retinal concentration of YC-001 for 3.5 months, to mitigate the constant biosynthesis of structurally unstable P23H opsin. Future efforts should be dedicated to either developing a retina-targeted delivery of YC-001 or safely increasing the half-life of a related active compound through medicinal chemistry. Once this goal is achieved, effects of the compound could be tested in the *P23H/*^*+*^ mouse model of adRP. It should be noted that rates of serum clearance in mice were recently reported for therapeutic GPCR ligands^59^ that are similar to the values of YC-001 clearance reported here. Significantly, these clearance rates were considerably slower for the same drugs in humans. Thus, YC-001 metabolism could differ significantly in humans versus mice.

In conclusion, we have identified a novel pharmacological chaperone as well as the first non-retinal modulator of rod opsin with both inverse agonist and non-competitive antagonist activities. Both the rod opsin antagonist activity and in vivo efficacy of YC-001 in a mouse model of bright light-induced retinal degeneration demonstrated the therapeutic potential of this compound in treating retinal degenerations associated with disrupted rhodopsin homeostasis.

## Methods

### Stable cell lines

U2OS(PLC-EA/P23H-PK) cells. A U2OS stable cell line was generated by DiscoveRx, Inc. (Fremont, CA, USA) for the β-Gal fragment complementation assay used in the small-molecule HTS for rescue of P23H-opsin transport^18^. U2OS cells that continually express P23H-PK and PLC-EA fusion proteins were generated^18^. Briefly, this small subunit of β-Gal (PK fragment) was fused to the C-terminal of the mouse P23H-opsin mutant, while the large EA fragment, a subunit of β-Gal, was fused with PLC, a plasma membrane-anchored peptide. Both constructs were subcloned by DicoveRx, Inc. and transferred into U2OS cells by viral infections. Positive clones were selected under treatment with hygromycin and geneticin. Expression of both fusion proteins was confirmed by immunostaining and immunoblots^18^.

Three NIH3T3 stable cell lines, NIH3T3-(WT-opsin/GFP), NIH3T3-(P23H opsin/GFP), and NIH3T3-(GFP) were previously generated which stably express mouse WT-opsin and GFP; P23H opsin and GFP; and only GFP, respectively^18,33^. GFP is expressed separately from opsin in these cell lines. NIH3T3 cells were from ATCC. DNA constructs for generating these stable cell lines were confirmed by Sanger sequencing. Expression of GFP was confirmed by green fluorescence imaging, and expression of P23H and WT-opsin were confirmed by immunoblots.

### Cell culture and media

Stable cell lines used in this study were cultured in Dulbecco’s modified Eagle’s medium (DMEM, Hyclone, Logan, UT, USA) with 10% fetal bovine serum (FBS; Hyclone) and 5 µg mL^−1^ Plasmocin antimycoplasma reagent (InvivoGen, San Diego, CA, USA) at 37 °C in 5% CO_2_. Cells were subcultured following the ATCC Animal Cell Culture Guide (www.atcc.org). When cells were seeded for assays in 96- or 384-well plates, they were cultured in medium containing 100 units mL^−1^ penicillin, 100 µg mL^−1^ streptomycin and 2.92 µg mL^−1^ l-glutamine (Hyclone) along with appropriate assay components as described below.

### Chemicals and reagents

DDM (Affymetrix Inc., Maumee, OH, USA) was used to solubilize bovine opsin from ROS disc membranes. The β-Gal fragment complementation assay was performed with the Gal-Screen System (Applied Biosystem, Bedford, MA, USA). PNGaseF was purchased from NEB (Ipswich, MA, USA) for the deglycosylation of cell lysates. 4′,6′-Diamidino-2-phenyl-indole (DAPI) and Hoechst33342 were purchased from ThermoFisherScientific (Grand Island, NY, USA) for nuclear staining. Cy3-conjugated goat anti-mouse secondary antibody was ordered from Jackson ImmunoResearch Laboratories, Inc. (catalogue number: 115-165-146, West Grove, PA, USA) for immunostaining. DMSO and 9-*cis*-retinal were obtained from Sigma-Aldrich Corp. (St. Louis, MO, USA). Mouse monoclonal B6-30 and 1D4 anti-rhodopsin antibodies were purified from hybridoma cells^60–62^. Alexa 488-conjugated B6-30 anti-rhodopsin antibody was obtained using the Alexa Fluor 488 Antibody Labeling Kit (ThermoFisherScientific). Forskolin was purchased from Tocris Biosciences (Bristol, UK). Scriptaid was purchased from Selleck Chemicals (Houston, TX, USA). YC-001 and its related compounds were synthesized and purified as described below under Medicinal Chemistry.

### Small-molecule libraries

To identify compounds rescuing the transport of P23H opsin, three chemical libraries were tested: the 25K University of Cincinnati Diversity Set, the 50K Life Chemicals Diversity Set, and the 2400 Spectrum Collection with pharmacological active compounds. A total of 79,080 compounds were tested for their activities with the β-Gal fragment complementation assay.

### β-Gal fragment complementation assay for HTS

To identify active compounds that rescue the transport of P23H opsin from the ER to the plasma membrane, we employed a small-molecule HTS using the β-Gal fragment complementation assay^1,15^. Briefly, U2OS(PLC-EA /P23H-PK) cells were suspended in assay medium at 3 × 10^5^ cells mL^−1^. The cell suspension was dispensed into 384-well plates (Greiner Bio-one, Monroe, NC, USA) at 20 µL per well, with a Multidrop dispenser (Thermo Scientific, Waltham, MA, USA). Plates with cells then were centrifuged at 200×*g* for 15 s before incubation at 37 °C with 5% CO_2_. Plates were removed 24 h later for treatment with tested compounds. A total of 248 plates containing 79,080 compounds from three small-molecule libraries were paired with an equal number of assay plates with cultured cells. Owing to a limited capacity, these plates were assayed in seven batches. An average of 56.3 nL of 10 mM compound in DMSO was dispensed into each well from column 3 to 22 of paired assay plates using a 50 nL 384-pintool system, such that the final concentration of each compound was 22.52 µM on average. To make the volumes of each well equal to 25 µL per well, columns 1, 3–22, and 24 were dispensed with 5 µL per well of assay medium, whereas columns 2 and 23 of each assay plate were dispensed with 5 µL per well of 0.5% DMSO and 5 µL per well of 25 µM 9-*cis*-retinal, as 0 and 100% controls, respectively. Each assay plate was shaken for 5 s before further incubation at 37 °C with 5% CO_2_. After 24 h, assay plates were removed, and each well was incubated with 25 µL of 1× Gal-Screen assay buffer (1:24 substrate-buffer ratio). Assay plates were incubated in the dark for 60–90 min, and luminescence was read from each assay plate with an Envision microplate reader (PerkinElmer) during a 0.1 s per well integration time. The mean and s.d. of readouts in column 2 of each plate were calculated as Mean_0%_ and SD_0%_, whereas the average and s.d. of readouts in column 23 were calculated as Mean_100%_ and SD_100%_. Eq. 1: Activity scores (%) = (RLU_cp_ − Mean_0%_)/(Mean_100%_ − Mean_0%_) × 100%, where RLU_cp_ is the relative luminescence unit derived for cells treated with a compound. Eq. 2: *Z*′-factor = 1–3 × (SD_0%_ + SD_100%_)/(Mean_100%_ − Mean_0%_)^32^; Eq. 3: signal-to-background (S/B) ratio = Mean_100%_/Mean_0%_. Activities of all tested compounds were registered with their compound ID by GeneData Screener (Genedata, Basel, Switzerland) and Pipeline Pilot software (Accelrys, San Diego, CA, USA). Hits, cherry-picked from a separate stock dose-dependent activity test, were selected with an activity score cutoff at 20%. Activity of each hit was tested again with the β-Gal fragment complementation assay in a 10-dose series with each dose tested in triplicate. The 10 doses tested were 80, 40, 20, 10, 5, 2.5, 1.25, 0.625, 0.313, and 0.156 µM. Dose-dependent curves of each hit compound were fitted with GeneData and Origin software.

### Immunostain and image-based analyses of P23H-mutant opsin

To characterize the effect of active compounds on the localization of P23H-mutant opsin in mammalian cells, an image-based assay was used with cells immunostained for WT or P23H-mutant opsin protein^18^. Briefly, NIH3T3-(P23H opsin/GFP) or NIH3T3-(WT-opsin/GFP) cells were seeded at 5000 cells per well in a 384-well cell-carrier plate (PerkinElmer) and incubated at 37 °C with 5% CO_2_ for 2 h. Cells were treated with compounds as described for the β-Gal fragment complementation assay and incubated in assay medium for 24 h. The assay medium then was aspirated, and cells were fixed with 4% paraformaldehyde at 20 µL per well for 20 min at RT. Cells were immunostained in one of two ways: cell membranes were either permeabilized with 0.1% Triton X-100 for 15 min or left intact and then were incubated with 10% goat serum (Life Technologies). To detect opsin on the cell plasma membrane only, intact cells were immunostained with 20 µL per well of 20 µg mL^−1^ B6-30 anti-rhodopsin antibody that recognizes the N-terminal epitope on the extracellular side of rhodopsin. To detect total opsin, cells were permeabilized with Triton X-100 and immunostained with 50 µg mL^−1^ 1D4 anti-rhodopsin antibody specific for the C-terminal epitope on the intracellular side of rhodopsin. Opsin immunostaining was visualized by incubating cells with 5 µg mL^−1^ Cy3-conjugated goat anti-mouse IgG antibody. Three washes with phosphate-buffered saline (PBS: 10 mM Na_2_HPO_4_, 1.8 mM KH_2_PO_4_, pH 7.4, 137 mM NaCl, and 2.7 mM KCl) were performed between each step of incubation with antibody. In the last wash with PBS, DAPI was added to each well at 300 nM. Fluorescence images were obtained with the Operetta High Content Imaging System (PerkinElmer) using a ×20 long objective. Five fields were taken of each well for cell images using three channels for fluorescence: GFP (100 ms), Cy3 (300 ms), and DAPI (50 ms). Images were analyzed with Acapella software from the Columbus data storage and analysis system (PerkinElmer)^18^. For cells immunostained with opsin on the non-permeabilized cell membrane only, an average of total fluorescence intensity of Cy3 per cell was used to quantify opsin in each well of the 384-well plate. For cells immunostained with opsin in permeabilized whole cells, an average ratio of Cy3 intensity on the cell plasma membrane (PM) to that of the entire cell (opsin ratio PM-to-total) was calculated for each well of a 384-well plate to quantify opsin transport to the plasma membrane. The plasma membrane was defined within ± 5% of the cell border. Cell shapes were revealed by GFP fluorescence.

### Image-based analyses of Clarin-1-N48K-Venus

HEK 293 stable cells co-expressing human clarin-1 N48K-Venus fluorescent protein and DsRed-Express-DR fusion protein were obtained from Dr. Yoshikazu Imanishi at Case Western Reserve University (CWRU). Cells were cultured in DMEM medium with 10% FBS following guidelines from ATCC. To test effects of YC-001 treatments on clarin-1 N48K-Venus amounts in these cells, an image-based assay was performed^34^. Briefly, cells were seeded at 20,000 cells per well in 40 µL per well DMEM medium with 10% FBS in a 384-well PerkinElmer View plate coated with poly-d-lysine and cultured at 37 °C with 5% CO_2_ for 24 h. Cells then were treated with 10 µL per well DMEM medium with 10% FBS containing 5× the final concentration of tested compounds. Such compounds included: MG-132 (Selleckchem, Houston, TX, USA), YC-001 (synthesized), scriptaid (Selleckchem) and tunicamycin (Sellechem). Each compound was tested with 10 doses in a twofold dilution series, featuring three biological replicates. The 384-well plate was put back for incubation at 37 °C with 5% CO_2_. After 24 h of exposure to test compounds, the plate was removed, and the medium was aspirated and replaced by 20 µL per well of 4% paraformaldehyde for fixation at RT for 20 min. Cells then were washed once with 50 µL per well of PBS before adding 50 µL per well of PBS containing 10 µM of Hoechst33342 for nuclei staining. After 15 min of dark adaptation at RT, the plate was sealed with a transparent film and removed for imaging by an Operetta High Content Imager (PerkinElmer). Each well was imaged for four fields in each corner of the well with four channels including bright field, YFP, DsRed, and Hoechst33342. Images were analyzed by the Columbus storage and analysis system (PerkinElmer). Hoechst33342 fluorescence images were used to define nuclei and count cells. Bright field images were employed to define cells and select populations of intact cell images. YFP and DsRed fluorescence intensities per cell were measured in each well.

### Immunoblots and quantification

NIH3T3-(P23H opsin/GFP) or NIH3T3-(WT-opsin/GFP) cells were seeded in a 48-well plate (Corning Costar) at 3.2 × 10^5^ cells per well and incubated at 37 °C with 5% CO_2_ for 2 h. One hundred-µL of assay medium containing a compound at 5× its final concentration was added to each well. The plate was gently shaken for 5 s before further incubation at 37 °C with 5% CO_2_ for 24 h. The assay medium then was aspirated, and cells were lysed with 100 µL per well PBS containing 0.1% Triton X-100 and complete protease inhibitor cocktail (Roche, Basel, Switzerland) followed by sonication for 3 s. Protein concentrations were determined with the Bradford assay. For PNGaseF catalyzed deglycosylation, 3 µL of PNGaseF (1500 U, NEB) was added to the cell lysate and the mixture was incubated at room temperature for 1 h before immunoblotting. For NIH3T3-(P23H opsin/GFP) cells, 15 µg of total protein were loaded per well onto an SDS-polyacrylamide gel; whereas for NIH3T3-(WT-opsin/GFP) cells, 5 µg of total protein was loaded per well. Opsin protein was immunostained with 0.2 μg mL^−1^ horseradish peroxidase-conjugated 1D4 anti-rhodopsin antibody. Band intensities were measured with ImageJ software (http://imagej.nih.gov/ij/; National the Institutes of Health, Bethesda, MD, USA) and normalized to a glyceraldehyde 3-phosphate dehydrogenase (GAPDH) loading control. Full scans of immunoblotted membranes are shown in Supplementary Fig. 8.

### Preparation of opsin membranes

ROS membranes were isolated from bovine retinas under dim red light^63^. To remove membrane associated proteins, ROS membranes were washed with a hypotonic buffer composed of 5 mM *bis*-tris propane (BTP) and 1 mM EDTA, pH 7.5, followed by gentle homogenization with subsequent centrifugation at 25,000×*g* for 30 min. This procedure was repeated four times. The final membrane pellet was suspended in 10 mM sodium phosphate buffer, pH 7.0, and 50 mM hydroxylamine to a 3 mg mL^−1^ concentration of rhodopsin, placed on ice and illuminated with a 150 Watt bulb for 30 min. Membranes were pelleted by centrifugation at 16,000×*g* for 5 min and then washed four times with 10 mM sodium phosphate buffer, pH 7.0, and 2% bovine serum albumin followed by 4 washes with 10 mM sodium phosphate buffer, pH 7.0, and two washes with 20 mM BTP, pH 7.5, and 100 mM NaCl. The concentration of opsin was measured with a UV–visible spectrophotometer and quantified using the absorption coefficient *ε*_280 nm_ = 81,200 M^−1^ cm^−1^^64^.

### Ligand-opsin binding assay

ROS membranes containing opsin were suspended in buffer composed of 20 mM BTP, pH 7.5, and 100 mM NaCl at a final concentration of 2.5 µM. YC-001, scriptaid or 9-*cis*-retinal were added to these membranes and incubated for a total of 30 min at RT. Treatment conditions included: 5 µM YC-001; 5 µM scriptaid; 5 µM 9-*cis*-retinal; incubation with *x* µM YC-001 for 15 min followed by addition of 5 µM 9-*cis*-retinal for another 15 min (*x* = 2.5, 5, 10, 20, 40, 60, 80 µM); incubation with 5 µM YC-001 and 5 µM 9-*cis*-retinal together. In a separate experiment, opsin membranes were incubated with a mixture of YC-001 or scriptaid together with 9-*cis*-retinal. Membranes then were solubilized with 20 mM DDM for 10 min at RT and UV–visible spectra of these samples were measured. To follow the kinetics of isorhodopsin regeneration, opsin membranes were treated with 0, 20, or 60 µM YC-001 for 15 min at RT. The treated opsin membranes were solubilized with 20 mM DDM for 10 min and then treated with 5 µM 9-*cis*-retinal. UV–visible spectra of the samples were measured every 2 min until 2 h of reaction time at RT. Each condition was repeated three times. The time course of isorhodopsin regeneration was fitted by a second-order exponential decay and apparent half-lives were obtained at the time when reaction reached half of the end-point product.

Alternatively, opsin incubated with the above ligands was purified by 1D4-immunoaffinity chromatography. Solubilized membrane lysates were cleared by centrifugation at 16,000×*g* for 15 min, and the supernatants were incubated with 1D4-immunoaffinity resin (6 mg of 1D4 anti-rhodopsin antibody per mL resin) equilibrated with 20 mM BTP, pH 7.5, 100 mM NaCl, 2 mM DDM for 1 h at RT. After washing, opsin samples were eluted by addition of 1D4 peptide (TETSQVAPA) to the above buffer and their spectra were measured with a UV–visible spectrophotometer.

To test if YC-001 could replace the 11-*cis*-retinal chromophore in rhodopsin, freshly isolated ROS membranes (2.5 µM) were treated with 20 µM YC-001 for 60 min, and absorption spectra were taken before and after treatment. An absorption spectrum of 100 µM YC-001 in 20 mM BTP, pH 7.5, and 100 mM NaCl was used as the control.

To test whether YC-001 stabilizes rod opsin, opsin samples (2.5 µM) were incubated with or without 10 µM YC-001 for 15 min, followed by solubilization in 20 mM DDM. Solubilized opsin samples were incubated at RT for 0, 1, 3, and 6 h before incubation with 10 µM 9-*cis*-retinal in the dark for isorhodopsin regeneration. UV–visible spectra of these samples then were measured.

### Fluorescence spectroscopy

To confirm the binding of YC-001 in the retinoid-binding pocket of rod opsin, quenching of Trp residues was monitored before and after adding increasing concentrations of YC-001, scriptaid or 9-*cis*-retinal ligands to ROS membranes containing opsin. Emission spectra were recorded with a PerkinElmer L55 Luminescence Spectrophotometer at 20 °C between 300 and 450 nm after excitation at 295 nm, with excitation and emission slit bands set at 5 and 10 nm, respectively. Changes in Trp fluorescence (Δ*F/F*0; where Δ*F* is the difference between the initial Trp fluorescence recorded at 330 nm (*F*0) and Trp fluorescence recorded at 330 nm at a specified YC-001 concentration) were plotted as a function of the ligand concentration. Binding curves were fitted by the Hill function using Origin software for each compound and the EC_50_ was calculated. All experimental data were corrected for background and self-absorption of excitation and emission light (inner filter effect).

### Bovine opsin crystallization for raman spectroscopy

Bleached ROS membranes bearing opsin were solubilized with either 20 mM BTP, pH 7.5, or 50 mM MES, pH 6.4, together with 130 mM NaCl, 1 mM MgCl_2_, 10% sucrose, and 1% *n*-octyl-β-d-glucopyranoside (OG) for 1 h at 4 °C. Insoluble debris was removed by centrifugation at 16,000×*g* for 5 min at 4 °C. Crystallization screens were performed by the sparse matrix crystallization method^65^ based upon previously published crystallization conditions for rhodopsin and opsin^25,66^. Each hanging drop was prepared by mixing equal volumes of solubilized opsin and a reservoir solution containing 3.0–3.6 M ammonium sulfate in 0.05–0.1 M sodium acetate buffer, pH 5.2–5.6. Crystals appeared within 2–5 days at 4 °C and were analyzed directly by Raman spectroscopy.

### Raman spectroscopy

Here, we used Raman microscopy to test if YC-001 binds rod opsin in a single crystal. A rod opsin crystal was transferred into 4.5 µL of fresh reservoir solution on a siliconized glass coverslip and transferred into a hanging drop crystallization tray where the well contained 1 mL of the same solution. An 80 mW, 647.1 nm Kr^+^ laser beam (Innova 70 C, Coherent, Palo Alto, CA, USA) was focused on the rod opsin crystal with a ×20 objective. The Raman spectrum of the rod opsin crystal was accumulated over 100× 1 s. The laser beam was then focused on the drop around the crystal, and a Raman spectrum for the holding solution was acquired and subtracted from the spectrum of the opsin crystal. To test whether YC-001 binds to the rod opsin, 0.5 µL of 100 mM YC-001 in DMSO was added to a 4.5 µL drop surrounding a rod opsin crystal. The opsin crystal was soaked with YC-001 for about 20 min to reach equilibrium, and then a Raman spectrum of the same opsin crystal was acquired for 100× 1 s. In parallel, a Raman spectrum of the surrounding solution containing YC-001 was collected and subtracted from the spectrum of the YC-001 soaked crystal. To obtain the Raman difference spectrum, a secondary subtraction was performed as Eq. 4: Raman difference spectrum = [Spectrum_(YC-001 soaked opsin crystal)_ − Spectrum_(surrounding solution with YC-001)_] − [Spectrum_(rod opsin crystal)_ − Spectrum_(surrounding solution)_]. To obtain the YC-001 standard spectrum, a Raman spectrum of the surrounding solution was subtracted from the spectrum of 10 mM YC-001 in the surrounding solution. Because YC-001 was added to the crystal using DMSO as dissolving solution that is absent in the opsin crystal, the difference spectrum showed a peak at 1420 cm^−1^ derived from DMSO.

### cAMP quantification assay

NIH3T3-(Opsin/GFP) and NIH3T3-(GFP) were plated in two 96-well plates at a density of 50,000 cells per well in 100 µL of DMEM medium containing 10% FBS and antibiotics. After 24 h, cells were washed with Krebs Ringer bicarbonate buffer containing glucose (KRBG), and incubated with KRBG buffer containing 100 µM cAMP specific phosphodiesterase inhibitor, Ro 20–1724 (Tocris, UK) at RT for 10 min. Under dim red light, cells then were treated with 25 µL of 6× its final concentration of forskolin (final 20 µM) followed by addition of 25 µL 6× final concentration of 9-*cis*-retinal, YC-001 or 9-*cis*-retinal and YC-001 together. While one plate then was wrapped with aluminum foil, the second plate was exposed to regular room light. Both plates were kept in a cell culture incubator for 15 min at 37 °C in 5% CO_2_. Levels of accumulated cAMP were detected with the Catchpoint cAMP fluorescent assay kit (Molecular Devices, Sunnyvale, CA USA) and the fluorescence with excitation/emission at 530/590 nm was read with a Flexstation3 plate reader (Molecular Devices) as described in the manufacturer’s protocol.

### *G*_*t*_ activation assay

*G*_*t*_ was extracted and purified from frozen bovine ROS membranes as described in Preparation of Opsin Membranes. The intrinsic increase in the fluorescence from Gtα was measured with a L55 luminescence spectrophotometer (PerkinElmer Life Sciences) using an excitation and emission wavelengths of 300 and 345 nm, respectively^39,40^. To test the effect of YC-001 on the basal activity of rod opsin, opsin membranes were incubated for 15 min at 20 °C with a 40 µM concentration of either YC-001, the non-active analog-YC-014, 9-*cis*-retinal or a co-treatment with 9-*cis*-retinal and YC-001. DMSO-treated opsin membranes were used to obtain a baseline for the basal activity of opsin. The molar ratio of opsin to *G*_*t*_ was 1:10, with opsin at a concentration of 100 nM and *G*_*t*_ at 1000 nM. Opsin membranes treated with either 9-*cis*-retinal alone or a co-treatment of 9-*cis*-retinal and YC-001 were bleached for 1 min with a fiber light source (Dolan Jenner Industries Inc., Boxborough, MA) equipped with a 480 to 520 nm band-pass wavelength filter (Chroma Technology Corporation, Bellows Falls, VT, USA). This step was followed by the addition of 300 μM GTPγS (Sigma-Aldrich) to determine the GTPγS-induced complex dissociation and corresponding fluorescence changes. *G*_*t*_ activation rates were determined and plotted for the first 100 s of the *G*_*t*_ activation assay^40,67^.

### Animal care and treatment conditions

*Abca4*^*−/−*^*Rdh8*^*−/−*^^42^ mice with a 129 Sv/Ev or C57BL/6 mixed background were used for light-induced retinal degeneration assays. *Abca4*^*−/−*^*Rdh8*^*−/−*^ mice were genotyped to confirm that they did not carry the *Rd8* mutation but did carry the Leu variation at amino acid 450 of RPE65^68^. Male and female C57BL/6 mice (Jackson Laboratory, Bar Harbor, ME) at 6 weeks of age were used to test the effects of YC-001 treatment on the retinoid cycle and bw as well as to determine parenterally administrated YC-001 entering the eyes. YC-001 was dissolved in DMSO at 80–160 mg mL^−1^, and the solution was provided to mice by i.p. injection. All mice were housed and maintained in a 12 h light (≤10 lux)/12 h dark cycle in the Animal Resource Center at the School of Medicine, CWRU. Animal procedures and experimental protocols were approved by the Institutional Animal Care and Use Committee at CWRU and conformed to recommendations of both the American Veterinary Medical Association Panel on Euthanasia and the Association for Research in Vision and Ophthalmology.

### Bright light-induced retinal degeneration

Retinal degeneration was initiated by exposing *Abca4*^*−/−*^*Rdh8*^*−/−*^ mice for 30 min to white light with an intensity of 10,000 lux (150-W spiral lamp, Hampton Bay, Home Depot, Atlanta, GA USA)^43^. Pupils of mice were dilated with 1% tropicamide 3 min before bright light exposure. YC-001 or DMSO were administered i.p. 30 min before such exposure. The effects of YC-001 were tested at two dosages: 50 and 200 mg kg^−1^ bw. The volume of each injection was less than 50 µL. Retinal structures were analyzed by SD-OCT seven days after bright light exposure. Mice were then euthanized, and their eyes were subjected to HE staining and microscopic imaging.

### SD-OCT imaging

To assess the effect of YC-001 treatment on *Abca4*^*−/−*^*Rdh8*^*−/−*^ mice following bright light-induced retinal degeneration, we performed ultrahigh-resolution SD-OCT (Bioptigen, Morrisville, NC) for in vivo imaging of mouse retinas^43^. Briefly, pupils of mice were dilated with 1% tropicamide. Three min later, mice were anesthetized by i.p. injection of a cocktail containing ketamine (20 mg mL^−1^) and xylazine (1.75 mg mL^−1^) at a dose of 4 μL g^−1^ bw. The A scan/B scan ratio was set at 1200 lines. Four frames of OCT images scanned at 0° were acquired in the B-mode, averaged, and saved as PDF files. To measure changes to photoreceptors in the retinas challenged with bright light and assess the effect of YC-001 on retinal protection, the thickness of the ONL was measured along the scanned SD-OCT image at 8 points from the nasal to temporal end of the retina. Each treatment group contained three mice, and a graph of ONL thicknesses was plotted to obtain the means and s.d.s of the triplicate samples.

### Retinal histology after HE staining

To examine the overall structure of the retinas subjected to bright light and treatment with YC-001, mice were euthanized, and their eyes were removed and fixed in 4% paraformaldehyde and 0.5% glutaraldehyde before paraffin sectioning. Paraffin sections (5 μm thick) were stained with HE and imaged by light microscopy (Leica, Wetzlar, Germany).

### Quantification of YC-001 in mouse eye by HPLC

To measure the amount of YC-001 in the eye following systemic delivery, YC-001 was administered to C57BL/6 mice by i.p. injection at a dose of 200 mg kg^−1^ bw. Mice then were euthanized at 30 min, 3 h and 24 h after these injections, and their eyes were removed for analysis. Eyes from two mice under the same treatment were homogenized on ice in 1 mL of PBS:methanol (1:1 ratio). Four-mL of hexanes were then added to the homogenized sample, and the mixture was vortexed for 15 s. The mixture was centrifuged at 3,220×*g* for 15 min at 4 °C to separate the hexanes from the aqueous layer. From the top hexane layer, 3.5 mL was transferred to a glass vial. This sample was then dried in a Savant speed vacuum concentrator (Thermofisher, Waltham, Massachusetts, USA) and dissolved in 300 µL methanol. One hundred-µL of dissolved sample were injected into an HPLC system connected to an Agilent Sil column (5 μm, 4.6 × 250 mm; Agilent Technologies, Santa Clara, CA) for separation with 10% ethyl acetate in hexanes at a flow rate 1.4 mL min^−1^. A chromatogram of absorption at 340 nm was then obtained. A YC-001 standard was subjected to the same procedures as the ocular samples and used to establish a retention time at 13.2 min. By comparing ocular samples to its standard curve of YC-001, the amount of YC-001 was then quantified in pmol per eye at different time points after its systemic administration. The identity of YC-001 in the peak fraction with a retention time of 8.8 min was confirmed by LC–MS using the same chromatography method as applied for HPLC.

### Synthesis of YC-001

**Fig. 9:**
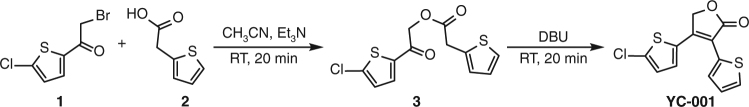
Synthesis of YC-001. YC-001 was synthesized by a two-step reaction. The 2-bromo-1-(5-chlorothiophen-2-yl)ethan (**1**) and 2-(thiophen-2-yl)acetic acid (**2**) were condensed in the presence of trimethylamine(Et_3_N) and acetonitrile (CH_3_CN) at room temperature (RT) for 20 min yielding the 2-(5-chlorothiophen-2-yl)-2-oxoethyl 2-(thiophen-2-yl)acetate (**3**), which was then treated with 1,8-diazabicycolo[5.4.0]undec-7-ene (DBU) at RT for 20 min yielding the target compound, YC-001

YC-001 was initially obtained from the University of Cincinnati for activity confirmation in mammalian cells. Its chemical structure was confirmed by nuclear magnetic resonance (NMR) spectroscopy and LC–MS (Supplementary Data 6). To supply enough compound for in vivo studies, YC-001 was synthesized as described in Fig. 9. Condensation of 2-bromo-1-(5-chlorothiophen-2-yl)ethan (**1**) and 2-(thiophen-2-yl)acetic acid (**2**) in the presence of trimethylamine, followed by treatment with 1,8-diazabicycolo[5.4.0]undec-7-ene (DBU) yielded the target compound, YC-001.

### COX-1 activity assay

The effect of the compound YC-001 on COX-1 activity was evaluated using the COX-1 Inhibitor Screening Kit (Abcam, Cambridge, MA, USA) according to the manufacture’s protocol. Briefly, the assay is based on the fluorometric detection of Prostaglandin G2, the intermediate product generated by the COX-1 enzyme. COX reaction mix included ovine COX-1, COX probe, and COX cofactor was prepared in a 96-well plate. YC-001 working solutions were dissolved in DMSO, and then mixed with COX Reaction Mix to the final concentrations (80, 40, 20, 10, 5, 2.5, 1.25, 0.625, 0.312, 0.156, 0.078 and 0.039 µM). Equivalent volume of DMSO was used as vehicle control and SC560 was used as a positive control. All controls and samples were measured in triplicate. Then arachidonic acid was added into each well to initiate all the reactions at the same time. The fluorescence was measured at Ex/Em = 535/587 nm in a kinetic mode for 15 min at 25 °C. Five time points were chosen in the linear range of the plot and the corresponding values for the fluorescence were used to calculate the slope of the linear regression equation. Relative COX-1 activity was calculated as Eq. 5: ${\mathrm{Relative}}\;{\mathrm{COX}} - 1\;{\mathrm{Activity}}\;(\% ) = \frac{{{\mathrm{Slope}}\;{\mathrm{of}}\;{\mathrm{sample}}}}{{{\mathrm{Slope}}\;{\mathrm{of}}\;{\mathrm{enzyme}}\;{\mathrm{control}}}} \times 100$.

### Metabolic stability of YC-001

A standard metabolic stability assay was performed in the presence of mouse or human liver microsomes. YC-001 (5 µM) was incubated with the microsomes (0.125 mg mL^−1^) resuspended in 0.2 mL of PBS buffer, pH 7.4 composed of 10 mM Na_2_HPO_4_, 1.8 mM KH_2_PO_4_, 137 mM NaCl, and 2.7 mM KCl. The enzymatic reaction to produce oxidized metabolites was initiated by addition of NADPH (1 mM). Samples were incubated at 37 °C for up to 240 min. Incubation without the cofactor was conducted in parallel to assess NADPH-independent clearance. The reactions were stopped with 0.2 mL of methanol followed by 0.3 mL chloroform. Residual YC-001 was extracted by vigorous shaking. To facilitate phase separation, the samples were spun down for 2 min, 15,000×*g*. The chloroform fraction was collected, dried down, and the extracted organic compounds were redissolved in 0.2 mL of methanol. To quantify YC-001, the samples were injected onto an Eclipse XDB-C18 column (4.6 × 150 mm, 5 µm) (Agilent Technologies) equilibrated with solvent composed of 30% acetonitrile in water (v/v), and 0.1% formic acid. YC-001 was eluted in a gradient of acetonitrile in water (30–100%) developed within 15 min at a flow rate of 1 mL min^−1^, detected at 350 nm, and quantified by correlating peak areas with known quantities of an original synthetic standard.

To assist in the interpretation of the YC-001 metabolic clearance data, two benchmark compounds verapamil (2-(3,4-dimethoxyphenyl)-5-[2-(3,4-dimethoxyphenyl)ethyl-methylamino]-2-propan-2-ylpentanenitrile) (Sigma-Aldrich) and quinidine ((S)-[(2R,4S,5R)-5-ethenyl-1-azabicyclo[2.2.2]octan-2-yl]-(6-methoxyquinolin-4-yl)methanol) (Sigma-Aldrich) were in the same experimental conditions as YC-001. The enzymatic reactions were stopped by addition of 0.3 mL ice-cold acetonitrile. To enable mass spectrometry-based detection and quantification of these drugs, 1 nmol of internal standard d3-verapamil (Cayman Chemical Company, Ann Arbor, MI, USA) for verapamil or imipramine (3-(5,6-dihydrobenzo[b][1]benzazepin-11-yl)-*N*,*N*-dimethylpropan-1-amine) (Alfa Aesar, Haverhill, MA, USA) for quinidine were added to the samples before they were spun down (15 min at 15,000×*g*) and injected onto a C18 X-Bridge column (100 × 2.1 mm; 3.5 μm) (Waters, Milford, MA, USA). HPLC separation of verapamil, quinidine, and the internal standards was achieved by a linear gradient of acetonitrile from 30 to 100% in water (v/v) developed within 15 min at a flow rate of 0.5 mL min^−1^. All solvents contained 0.1% formic acid (v/v). The HPLC eluate was sprayed into a LXQ linear-trap mass spectrometer (ThermoFisherScientific) via an electrospray probe operating in the positive ionization mode. Parameters of ionization and detection were tuned with synthetic standards for these drugs to achieve the highest possible sensitivity. Verapamil and its deuterated form were detected by selected reaction monitoring (SRM) using *m*/*z* 455.3 → 303.2 and 458.3 → 306.3 transitions, whereas quinidine and its corresponding internal standard (imipramine) were detected by fragmentation at *m*/*z* 326.3 → 307.2 and 281.2 → 86.1, respectively. For quantification, calibration curves were generated based on the linear relationship between ratios of the SRM ion intensities corresponding to the drug and the internal standard versus the molar ratios of these compounds.

### Pharmacokinetics of YC-001 in mice

C57BL/6J mice ranging from 8 to 12 weeks of age were purchased from Jackson Labs. For administration, YC-001 was dissolved in DMSO at 160 mg mL^−1^. To obtain the blood clearance curve for YC-001, each animal was treated by i.p. injection of YC-001 at 200 mg kg^−1^ bw. After administration, blood was collected at 5, 10, 15, 20, 30, 45, 60, and 90 min from the orbital sinus and dropped into K_2_EDTA blood collection tubes (FisherScientific). Mice were anesthetized with a ketamine/xylazine cocktail two min before blood collection. The blood samples were then incubated at 4 °C for 2 h before centrifugation at 1800×*g* for 20 min to collect the plasma in the supernatant. Plasma was mixed with twice the volume of methanol and then stored at −80 °C. YC-001 was extracted from the samples (300 to 600 μL) with 0.6 mL chloroform. The chloroform fraction was collected, dried, and the extracted organic compounds were dissolved in 0.25 mL of methanol. One hundred µL of each sample was injected onto an Eclipse XDB-C18 column (4.6 × 150 mm, 5 µm) and YC-001 was detected and quantified as described in the section Metabolic stability of YC-001.

### Micro Ames test for YC-001

To determine the potential genotoxicity of YC-001, a Micro Ames test was performed by the Charles River Laboratories, Inc. The S9 microsomal fraction was obtained from Moltox Molecular Toxicology, Inc. (Boone, NC, USA). A premixture was prepared including 25 µL of S9 mix (10% v/v S9 fraction in 8 mM MgCl_2_, 33 mM KCl, 100 mM sodium phosphate buffer, pH 7.4, +S9) or phosphate buffer (0.2 M sodium phosphate, pH 7.4, no S9), 5 µL of bacterial culture (>1000 × 10^6^ bacteria mL^−1^) and 100 µL of molten top agar supplemented with 0.05 mM biotin and minimal histidine (0.05 mM) and minimal tryptophan (0.05 mM). The 24-well plates were prepared by adding 1.3 mL of minimal bottom agar (1.3% agar, Vogel-Bonner medium E and 0.25% glucose) to each well. A 10 µL aliquot of YC-001 working solution/negative/positive control was added to each well followed by addition of 130 µL of the premixture. The plates kept on a leveled surface for 1 h while the top agar solidified, then were incubated at 37 °C for 72 h. After this period, plates were stored at 4 °C before revertant colony counts were manually recorded using an inverted microscope. A total of five bacterial strains were tested including *S. typhimurium* TA1535 *his*G46 *rfa* Δ*uvr*B (T1535), *S. typhimurium* TA97a *his*O1242 *rfa ΔuvrB* pKM101(TA97a), *S. typhimurium* TA98 *his*D3052 *rfa ΔuvrB* pKM101 (TA98), *S. typhimurium* TA100 *his*G46 *rfa ΔuvrB* pKM101 (TA100), and *E. coli* WP2 *trp uvrA* pKM101 (WP2). For each bacterial strain YC-001 was tested at a total of eight dosages in duplicate: 250, 75, 25, 7.5, 2.5, 0.75, 0.25, and 0.075 µg per well, with or without S9 metabolism. Without S9, positive controls for each bacterial strain were as follows: 0.05 µg per well sodium azide (NaAz) for TA1535 and TA100, 2 µg per well 9-aminoacridine hemihydrate (9-AC) for TA97a, 0.2 µg per well 2-nitrofluorene (2NF) for TA98, 0.1 µg per well 4-nitroquinoline N-oxide (NQO) for WP2. With S9, the positive controls for the five strains were: 0.1 µg per well 2-aminoanthracene (2AA) for TA1535, TA97a, TA98 and TA100, and 2 µg per well 2AA for WP2. Positive results (indicative of mutagenic potential) required both the following criteria: (1) The tested compound show more than 2 times the revertant colony counts than negative controls for TA100, TA97a and WP2 or more than three times for TA98 and TA1535; (2) the increased revertant colony counts reveal a concentration dependence. Negative results (not indicative of mutagenic potential) required the revertant colony counts of tested compounds to be less than two times of negative controls for TA100, TA97a, and WP2 or less than three times for T98 and TA1535.

### Cell-surface immunostain for rhodopsin mutants

Human rhodopsin cDNA was placed into the pcDNA3.1(+) vector (pcDNA3.1-hOpsin). Site-directed mutagenesis was performed following the QuickChange II site-directed mutagenesis kit (Agilent Technologies Inc.). Primers for the following mutations were as follows: T4R-forward, 5′-atgaatggcagagaaggccctaacttctacg-3′; T4R-reverse, 5′-cgtagaagttagggccttctctgccattcat-3′; P53R-forward, 5′-gctgggcttccgcatcaacttcctcacgc-3′; P53R-reverse, 5′-gcgtgaggaagttgatgcggaagcccagc-3′; G106R-forward, 5′-ggatacttcgtcttcaggcccacaggatgca-3′; G106R-reverse, 5′-tgcatcctgtgggcctgaagacgaagtatcc-3′; C110Y-forward, 5′-cgggcccacaggatacaatttggagggcttc-3′; C110Y-reverse, 5′-gaagccctccaaattgtatcctgtgggcccg-3′; D190N-forward, 5′-gctcgtgtggaatcaactactacacgctcaag-3′; D190N-reverse, 5′-cttgagcgtgtagtagttgattccacacgagc-3′; P267L-forward, 5′-gatctgctgggtgctctacgccagcgtggc-3′; P267L-reverse, 5′-gccacgctggcgtagagcacccagcagatc-3′. DNA vectors with rhodopsin mutations were confirmed by Sanger sequencing. After NIH3T3 cells were transfected with pcDNA3.1-hOpsin or its mutants for 24 h, these cells were resuspended and seeded into a 96-well plate with an optic bottom (Corning). Seeded cells were treated with YC-001(10 µM), 9-*cis*-retinal (5 µM) or DMSO (0.1% v/v). Each condition was repeated in three wells. The treated plate was covered in tin foil and incubated at 37 °C with 5% CO_2_ for 24 h. Cells were then immunostained with 50 µg mL^−1^ Alexa488-conjugated B6-30 anti-rhodopsin antibody. Cells were not exposed to detergents so that cell membranes were kept intact and only rhodopsin on the cell surface was immunostained. Alexa 488 fluorescence was imaged by the ImageExpress (Molecular Devices) high-content imager with a ×20 objective.

### Medicinal chemistry of YC-001

Analogs of YC-001 (YC-022 to YC-069) were synthesized by Charles River, Inc. (Wilmington, MA, USA). Detailed synthetic steps are described in Supplementary Note and Supplementary Fig. 9. LC–MS and NMR data of purified YC-001, and YC-022 to YC-069 are shown in Supplementary Data 6. YC-002-YC-021 were purchased from commercial vendors including Enamine LLC (Monmouth Jct., NJ, USA), Matrix Scientific (Columbia, SC, USA) and Tokyo Chemical Industry Co., Ltd. (Portland, OR, USA). Chemical purities of the YC compounds were higher than 94%, as determined by NMR and LC–MS. Activities of YC compounds were tested with the β-Gal fragment complementation assay, as described in a prior section. All YC compounds were tested at 10 doses, each in triplicate: 80, 40, 20, 10, 5, 2.5, 1.25, 0.625, 0.313, and 0.156 µM. Activity scores were standardized to the effect of 5 µM 9-*cis*-retinal under dark conditions. YC derivatives with efficacies higher than 20% to that of 9-*cis*-retinal were considered active compounds.

### Retinoid cycle analyses

To test if treatment with YC-001 affects visual pigment regeneration, we quantified the 11-*cis*-retinyl-oxime extracted from retinas of C57BL/6 mice after different periods of dark adaptation following exposure to bright light. Mice at 6 weeks of age were administered YC-001 at 200 mg kg^−1^ bw via i.p. injection. At 30 min following YC-001 administration, eyes were dilated with 1% tropicamide. Mice then were anesthetized with a cocktail containing 20 mg mL^−1^ of ketamine and 1.75 mg mL^−1^ of xylazine at a dose of 4 μL g^−1^ bw. Anesthetized mice were exposed to bright light with an intensity of 10,000 lux for 10 min to bleach about 90% of the rhodopsin pigment. Mice then were returned to the dark for recovery. Animals then were euthanized at 0, 2, 4, 6 or 24 h after bleaching and recovery in the dark, and their eyes were removed and homogenized in 1 mL of 1:1 PBS:ethanol mixture (v/v) containing 40 mM hydroxylamine. For each time point, three mice were used as replicates. Homogenized eye samples were incubated at RT for 20 min. In the dark, 4 mL of hexanes were added to each sample and the mixture was vigorously shaken for 2 min before centrifugation at 3200×*g* for 10 min, and transfer of the top hexanes phase to a glass tube. Hexanes were then evaporated in a speedvac concentrator for 30 min. Dried samples containing retinoids were suspended in 300 µL hexanes and transferred to a glass vial for HPLC analysis. 11-*cis*-Retinyl-oxime released from the regenerated rhodopsin pigments were separated on an Agilent Sil column (5 µm, 4.6 × 250 mm) with an isocratic flow of 10% ethyl acetate in hexanes (1.5 mL min^−1^) and detected at 325 nm. Amounts of 11-*cis*-retinyl-oximes per eye were quantified by normalizing the peak area to an 11-*cis*-retinyl-oxime standard. 11-*cis*-Retinyl-oximes were quantified in three eye samples per condition, and the means and s.d.s were calculated.

### ERG analyses

C57BL/6 mice at 2 months of age were placed in the dark overnight. Mice then were divided into a YC-001-treated group, a DMSO-treated group, and an unbleached group. Each group contained 3 mice (2 females and 1 male). Mice were given a single dose of YC-001 at 200 mg kg^−1^ by i.p. injection. The DMSO group was treated with an equivalent volume of DMSO compared to the YC-001-treated group. One h after YC-001 or DMSO administration, mouse eyes were treated with 1% tropicamide eye drops for pupil dilation, exposed to 2,000 lux of illumination for 5 min and returned to the dark. Mice were anesthetized after the bleach for scotopic ERG recordings^69^. Briefly, every 5 min a single-flash scotopic ERG at 1.6 cd s m^–2^ was recorded until 1 h after the bleach. A-wave amplitudes of each ERG recording were measured, averaged from three animals, and plotted as a function of time and fitted to a linear function using Origin software version 8.1.

### Body weight measurements

To estimate the long-term toxicity of YC-001 in mice, we administered YC-001 to C57BL/6 mice by daily i.p. injections from Day 14 to 38 after birth with one of two doses, either 100 mg kg^−1^ or 200 mg kg^−1^ bw. Equivalent volumes of DMSO were injected into control groups. Body weights of treated mice were measured daily. Each dosage group contained three mice including males and females. No deaths were observed either during the treatment phase or for 25 days following the indicated treatment.

### Statistics

Data collected for the β-Gal fragment complementation assay, image-based analyses, and the cAMP quantification assay included three biological replicates. Positive and negative controls were repeated 8 or 6 times, whereas compounds were tested in triplicate at six to ten concentrations. Effects of the tested compounds were analyzed in a dose-dependent manner to exclude random errors. For the opsin binding assay, isorhodopsin regeneration assay, compound stability assay and *G*_*t*_ activation assay, each experiment was repeated three individual times and parameters were averaged from those repeats with error bars as s.d.s. The effect of each compound was either plotted in a dose-dependent or time-dependent manner as compared with controls. All samples were included in the analyses.

For animal studies, two doses of YC-001 were used, with each dose tested in three animals including males and females. Age-matched animals were selected from the same one or two litters and were grouped randomly into different treatment conditions after ensuring that every group had at least one male and one female animal. Personnel who performed the retinal function, retinal imaging, or retinoid analyses were blinded as to the treatment status of these mice. All samples were included for animal studies. Sample size for the animal studies was validated by Gpower3 software using the post-hoc power analysis for a two-tailed *t*-test^70^. Effect size index was calculated in Eq. 6: $d = \frac{{\left| {\mu 1 - \mu 2} \right|}}{{\sqrt {0.5 \times \left( {\sigma 1^2 + \sigma 2^2} \right)} }} > 5$, that made the power (1 − *β* error probability) > 0.99 for sample size of 3. Average ONL thicknesses were *µ*1 and *µ*2 from SD-OCT of retinas treated with 200 mg kg^−1^ either YC-001 or DMSO, and *σ*1 and *σ*2 were s.d.s of those from YC-001- and DMSO-treated retinas, respectively.

### Data availability

Data generated by this study are included in the main text and Supplementary Files, and are available from the corresponding authors upon reasonable request.

## Electronic supplementary material

Supplementary Information

Description of Additional Supplementary Information

Supplementary Data 1

Supplementary Data 2

Supplementary Data 3

Supplementary Data 4

Supplementary Data 5

Supplementary Data 6
