# Supplementary material for: A novel small molecule chaperone of rod opsin and its potential therapy for retinal degeneration
Source: Nat Commun. 2018 May 17;9:1976. doi: 10.1038/s41467-018-04261-1 (PMC5958115; doi:10.1038/s41467-018-04261-1)
Supplement: Supplementary file 1 — Supplementary Information [file 41467_2018_4261_MOESM1_ESM.pdf]

A novel small molecule chaperone of rod opsin and its potential therapy for retinal degeneration

Yuanyuan Chen[1,2,7 *], Yu Chen[1,8], Beata Jastrzebska[1,3], Marcin Golczak[1,3], Sahil Gulati[1,3], Hong Tang[4], William Seibel[4], Xiaoyu Li[1], Hui Jin[1], Yong Han[5], Songqi Gao[1], Jianye Zhang[1], Xujie Liu[7], Hossein Heidari-Torkabadi[1], Phoebe L. Stewart[1,3], William E. Harte[6], Gregory P. Tochtrop[5], Krzysztof Palczewski[1,3 *]

[1] Department of Pharmacology, School of Medicine, Case Western Reserve University, 10900 Euclid Avenue, Cleveland, OH 44106, USA

[2] The McGowan Institute for Regenerative Medicine, University of Pittsburgh, 450 Technology Drive Suite 300, Pittsburgh, PA 15219, USA

[3] Cleveland Center for Membrane and Structural Biology, Case Western Reserve University, 1819 E 101st Street, Cleveland, OH 44106, USA

[4] Drug Discovery Center, University of Cincinnati, Cincinnati, 2180 E. Galbraith Road, Cincinnati, Ohio 45237

[5] Department of Chemistry, Case Western Reserve University, 10900 Euclid Avenue, Cleveland, OH 44106, USA

[6] Office of Translation and Innovation, Case Western Reserve University, 10900 Euclid Avenue, Cleveland, Ohio 44106, USA

[7] Present address: Department of Ophthalmology, University of Pittsburgh, 3501 Fifth Avenue, Pittsburgh, PA 15260, USA

[8] Present address: Yueyang Hospital and Clinical Research Institute of Integrative Medicine, Shanghai University of Traditional Chinese Medicine, Shanghai 200437, China

[*]Correspondence: Y.C. cheny1@pitt.edu and K.P. kxp65@case.edu

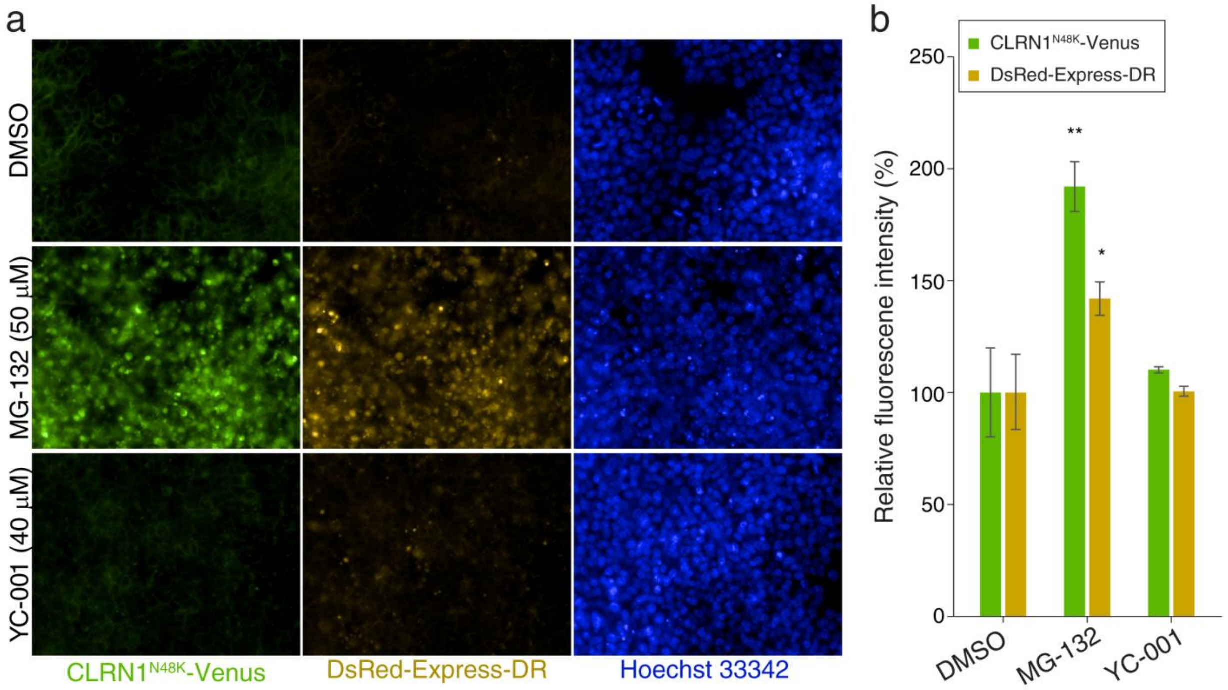

Supplementary Fig. 1: YC-001 does not stabilize clarin1^{N48K}-Venus. **a** Fluorescence images of HEK-293 cells expressing both CLRN1^{N48K}-Venus and DsRed-Express-DR treated with either DMSO (top), MG-132 (middle) or YC-001 (bottom). Left to right are fluorescence images of CLRN1^{N48K}-Venus, DsRed-Express-DR and Hoechst33342, respectively. **b** Relative fluorescence intensities of CLRN1^{N48K}-Venus (green bars) and DsRed-Express-DR from HEK-293 cells treated with DMSO, MG-132 or YC-001. Fluorescence intensities from DMSO-treated cells were normalized to 100%. Bars were averaged from sixteen biological replicates and standard deviations are shown as error bars. **, P=1.05 X 10^{-5}, *P=7.00 X 10^{-4} compared to DMSO-treated control, using a two-tailed Student test.

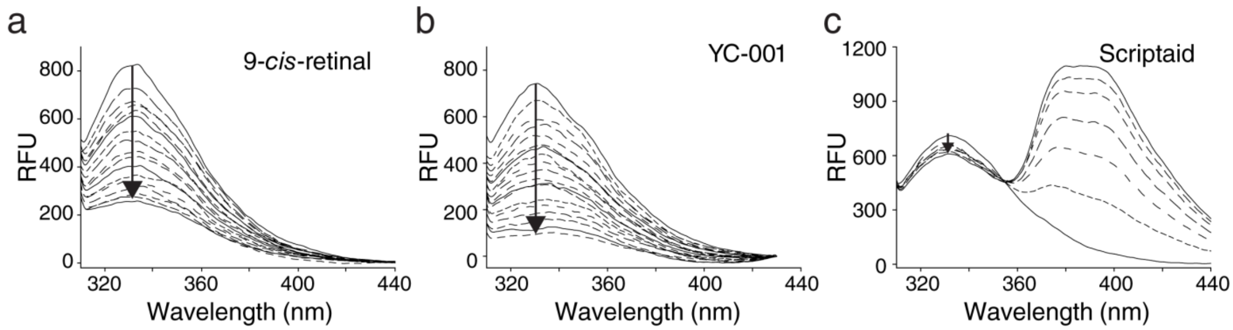

Supplementary Fig. 2: YC-001 binds to rod opsin in a manner that affects the chromophore-binding pocket. The Trp fluorescence spectra of opsin were recorded upon titration with different concentrations of either 9-*cis*-retinal (**a**), YC-001 (**b**) or scriptaid (**c**) in the dark. RFU, relative fluorescence units. Quenching of Trp opsin fluorescence by increasing concentrations of ligands is indicated with arrows. Changes of fluorescence intensity at 330 nm ($\Delta F/F0$) are plotted as a function of the concentration of 9-*cis*-retinal, YC-001 and scriptaid, respectively in Fig. 3e-g. The increased peak around 400 nm was due to scriptaid fluorescence. Each experiment was repeated three times.

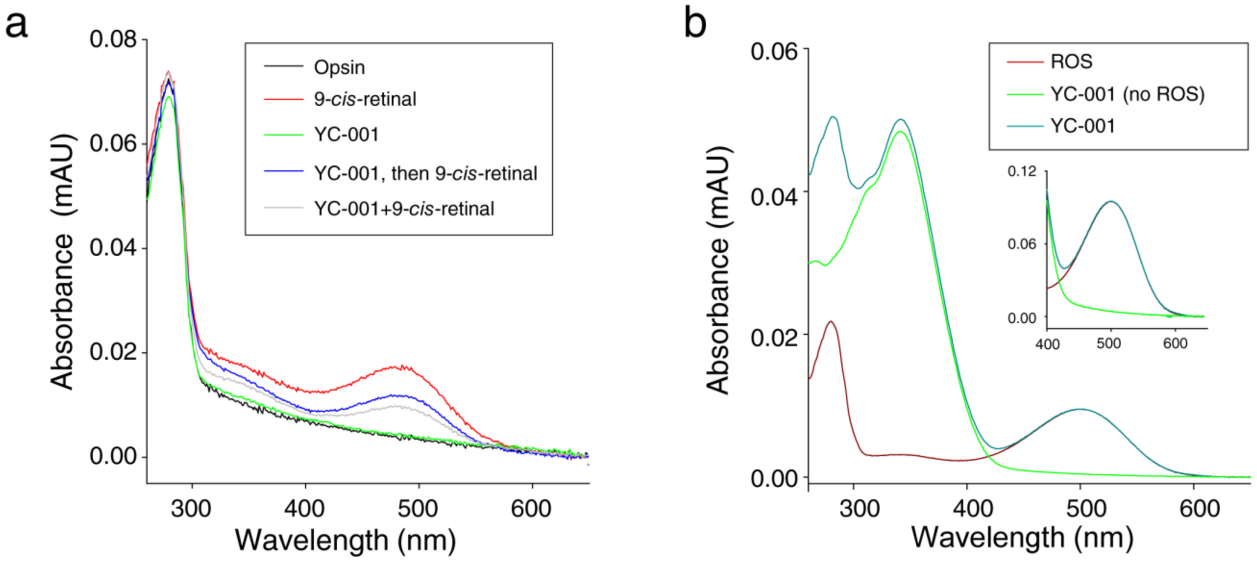

Supplementary Fig. 3: YC-001 binds to rod opsin through a non-covalent interaction. **a** Absorption spectra of isorhodopsin or opsin purified under conditions provided in Fig. 4 **(a,b**) by 1D4 immunoaffinity chromatography. **b** Absorption spectra of rhodopsin in ROS disc membranes under treatment with YC-001. Brown, ROS membranes; light green, YC-001 only; dark green, ROS disc membranes incubated with excess YC-001 for 90 min. Inset shows the enlarged region of the absorption spectra from 400 to 650 nm. Each experiment was repeated twice.

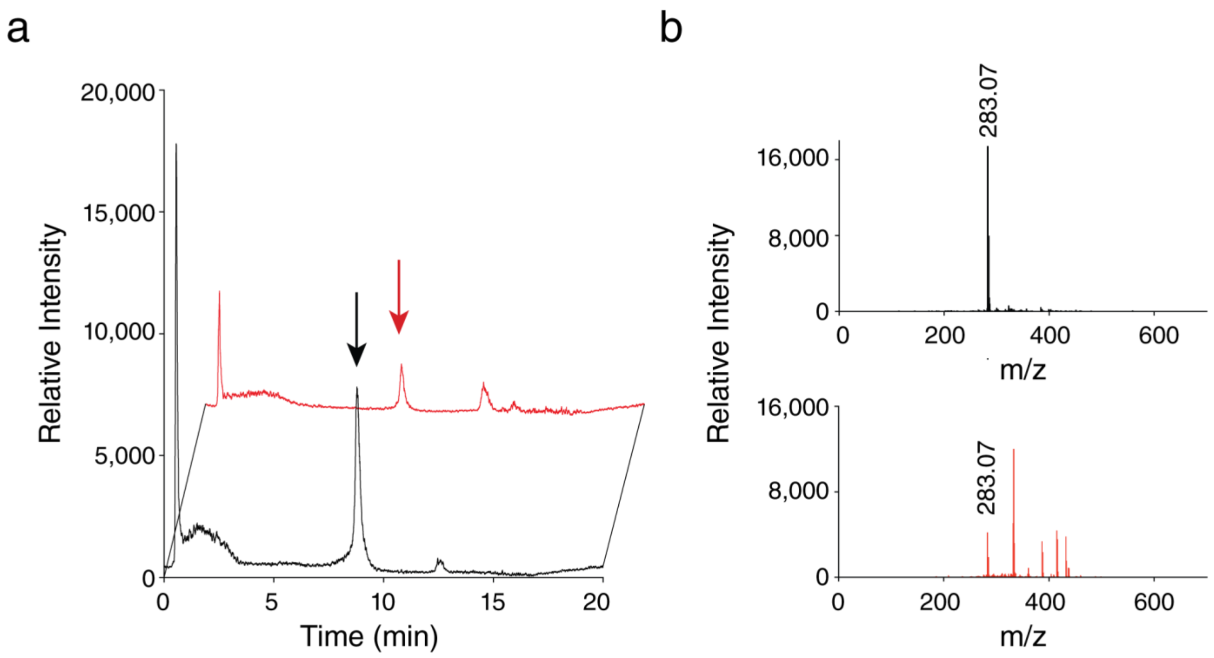

Supplementary Fig. 4: LC-MS analysis of YC-001 from mouse eyes. **a** Chromatogram of ions with m/z from 283 to 284. Black, YC-001 standard; red, extract from eyes of a mouse treated with YC-001, collected after purification with a retention time of 13.2 min (**Supplementary Fig. 4b**). **b** MS spectra at a LC retention time of 8.8 min (arrows in **a**). Top, YC-001 standard; bottom, mouse eye extract. The peak with m/z at 283.07 corresponds to YC-001. This experiment was repeated for twice.

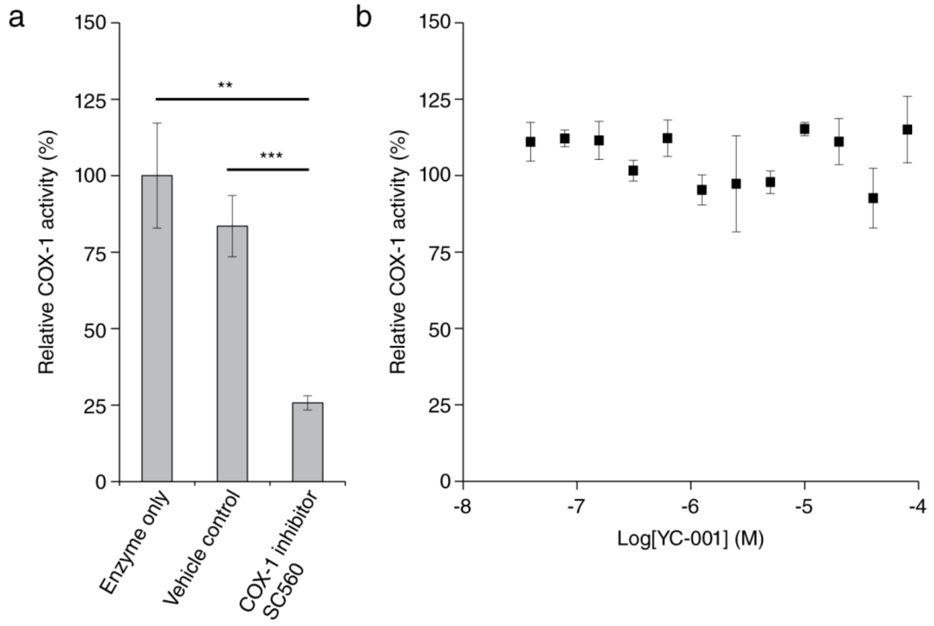

Supplementary Fig. 5: YC-001 does not affect the activity of COX-1. **a** Changes of relative COX-1 activity (% of Enzyme only) treated with DMSO as vehicle control and a COX-1 inhibitor, SC560, as positive control. Values and error bars indicate mean ± SD (n=3). ***p*<0.01 SC560 *versus* Enzyme only. ****p*<0.001 SC560 versus vehicle control. **b** The dose-response graph showed relative COX-1 activity (% of Enzyme only) treated with different concentrations of YC-001 in Log format. Relative COX-1 activities were normalized by the activity of Enzyme only as 100%. Values and error bars indicate mean ± SD (n=3). This experiment was repeated twice.

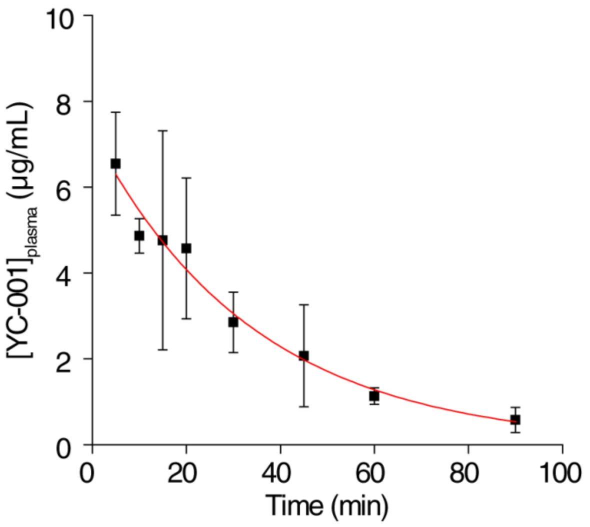

Name (Unit)	Dose (mg/kg)	Route	n	$T_{1/2}$ (min)	C_0 (μg/mL)	K_e (min^{-1})	V_d (L/kg)	Clearance (L/min/kg)
Value	200	i.p.	32	34.5	7.28	0.0201	27.5	0.552

Supplementary Fig. 6: Fast elimination of YC-001 in the plasma of C57BL/6 mice following intraperitoneal (i.p.) injection. The plot of plasma concentration of YC-001 ($[YC\text{-}001]_{plasma}$) *versus* time is shown in the top graph. $[YC\text{-}001]_{plasma}$ was measured at 5, 10, 15, 20, 30, 45, 60 and 90 min after administration via i.p. injection at 200 mg/kg body weight. Each data point and error bar was the average and standard deviation of $[YC\text{-}001]_{plasma}$ from four mice (2 female and 2 male) at 8-12 weeks of age, respectively. The elimination curve was fitted with the first-order exponential decay ($y=C_0 \times e^{(-x/T1/2)}$) using Origin Software. The bottom table shows the pharmacokinetic parameters estimated from the YC-001 elimination curve. $K_e = 0.693 / T_{1/2}$, $V_d = \text{Dose} / C_0$, and Clearance = $K_e \times V_d$. Dose, dose of YC-001 administered; Route, route of administration; n, total number of mice used for the plot; $T_{1/2}$, the half-life; C_0, the initial plasma concentration of YC-001 at time 0; K_e, estimated elimination rate constant; V_d, estimated volume of distribution. This experiment was performed once.

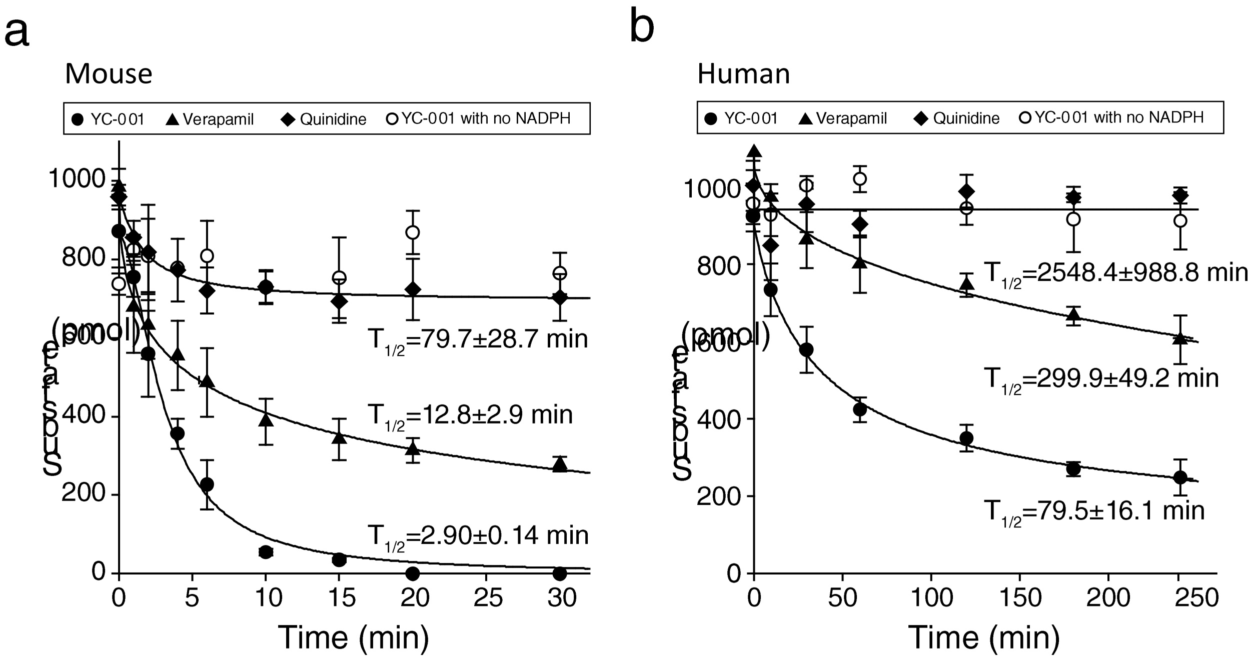

	Mouse liver microsomes		Human liver microsomes	
Compound	$T_{1/2}$ (min)	Cl_{int} (μL/min/mg)	$T_{1/2}$ (min)	Cl_{int} (μL/min/mg)
YC-001	2.9±0.14	1911.7	114.7 ± 23.2	48.33
verapamil	12.8±2.9	433.1	432.7 ± 71.0	12.81
quinidine	79.7±28.7	69.6	3676.5 ± 1426.5	1.51

Supplementary Fig. 7: YC-001is quickly cleared by mouse (**a**) or human liver microsomes (**b**). To predict the stability of YC-001 in the liver after *in vivo* treatment, 5 μM YC-001 was incubated with 0.125 mg/mL mouse or human liver microsomes and 1 mM NADPH for 30 or 250 min at 37 °C (solid circles). Verapamil (solid triangles) and Quinidine (solid diamonds) were tested under the same conditions, as rapid- and slow-clearance controls, respectively. YC-001 incubated with liver microsomes without NADPH was used as a negative control (open circles). The amount of each compound at different times was quantified by LC-MS. Each data point was obtained from an average of three biological repeats, with standard deviations of the repeats as corresponding error bars. Data from each compound was fitted to a first-order exponential decay with Sigmaplot software. Half-life ($T_{1/2}$) and initial Clearance (Cl_{int}) of each compound are listed in the bottom table. Cl_{int} (μL min^{-1} mg^{-1}) = 0.693X $1/T_{1/2}$ (min) X volume (μL) / mg. This experiment was repeated once with human and mouse liver microsomes.

Fig. 4a

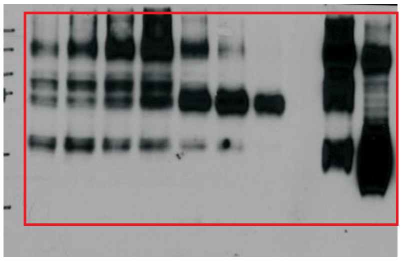

Fig. 4c

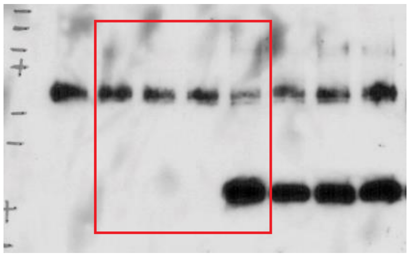

Fig. 4d

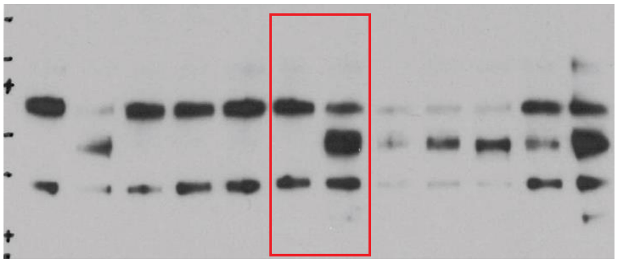

Supplementary Fig. 8: Full scans of immunoblotted membranes. Areas used in Fig. 4 are framed by red rectangles.

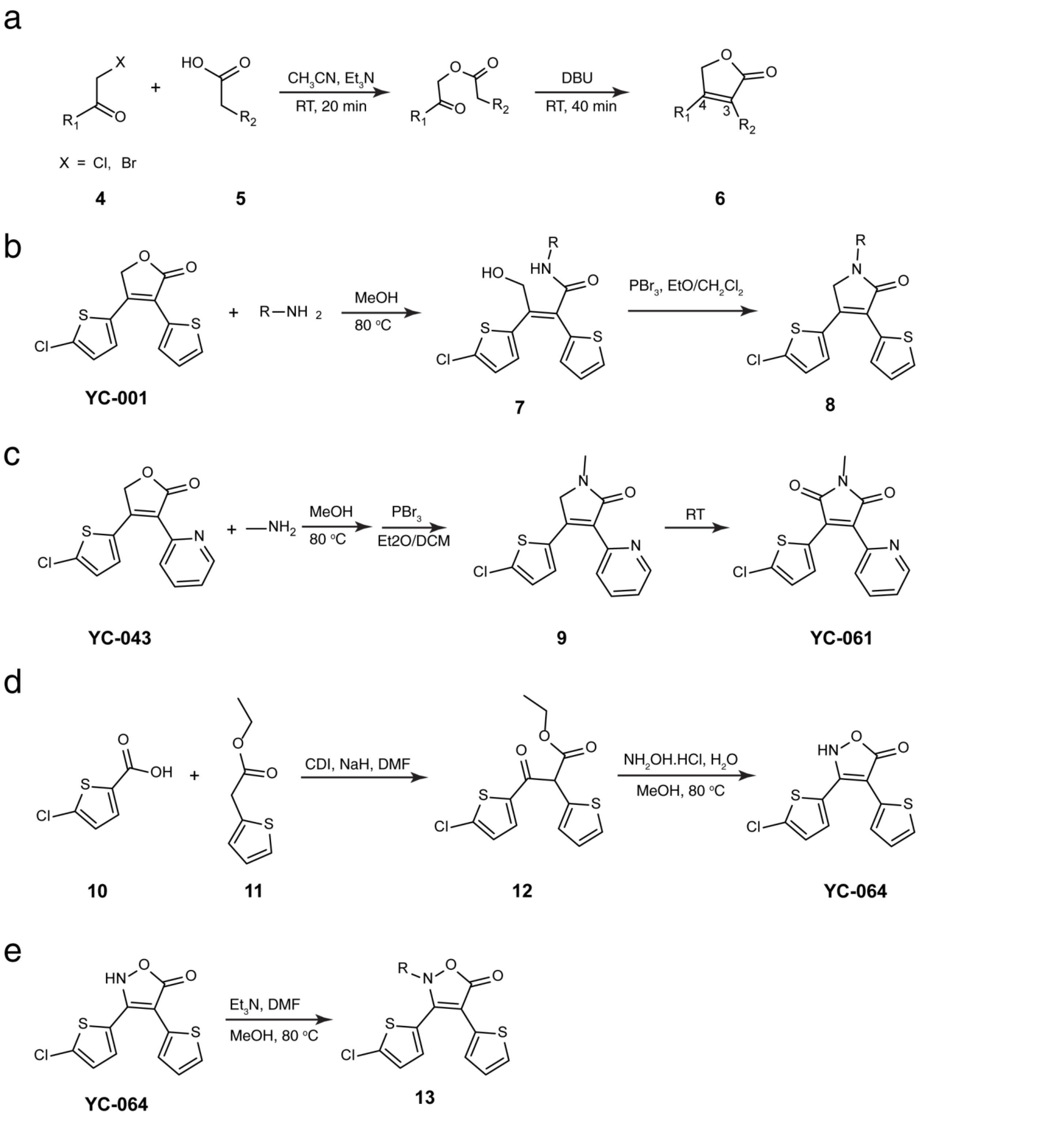

Supplementary Fig. 9: Synthesis of analogs of YC-001. **a** Synthesis of analogs of YC-001 with substitutions at C3 or C4 positions. X= Cl or Br. R1 and R2 are substitutions of subgroup I and III of YC-001 shown in Fig. 1c**.** Analogs of YC-001 were prepared by the condensation of α-halogeno ketones (**4**) with substituted acetic acids (**5**) in the presence of trimethylamine(Et3N) and acetonitrile (CH3CN) at room temperature (RT) for 20 min followed by intramolecular cyclization of the acetate intermediate with 1,8-diazabicycolo[5.4.0]undec-7-ene

(DBU), yielding the analogs of YC-001 (**6**). **b** Synthesis of analogs substituting the furan-2(5H)-one scaffold of YC-001 with a 1,5-dihydro-2H-pyrrol-2-one ring. Target compounds (**8**) were prepared by treatment of YC-001 with the appropriate amine (NH_2-R) neat or with methanol (MeOH) at 80 ^oC followed by intramolecular cyclization of the amide intermediate (**7**) with PBr_3 and ethylene oxide (Et_2O) or CH_2Cl_2. **c** Synthesis of YC-061. YC-043 was treated with methylamine in the presence of MeOH at 80 ^oC followed by incubation with PBr3 and Et_2O or CH_2Cl_2 to yield N-methyl analogue (**9**), which was then oxidized to YC-061 during purification and RT incubation. **d** Two-step synthesis of YC-064. A Claisen condensation of ethyl 2-(thiophen-2-yl)acetate (**11**) and (5-chlorothiophen-2-yl)imidazolide (**10**) produced the -ketoester (**12**) with carbonyldiimidazole (CDI), sodium hydride (NaH) and dimethylformamide (DMF). YC-064 was then obtained by cyclocondensation of hydroxylamine hydrochloride ($NH_2OH.HCl$) with -ketoester (**12**) in the presence of MeOH at 80 ^oC. **e** Synthesis of analogs replacing the furan-2(5H)-one scaffold of YC-001 with an isoxazol-5(2H)-one ring. Type **13** YC compounds were synthesized by N-alkylation of YC-064 with trimethylamine (Et_3N), DMF and MeOH at 80 ^oC.

Supplementary Table 1: Summary of compounds' effects on the rescue of P23H opsin transport by a HTS with the β-Gal fragment complementation assay. Activity scores are standardized to the effect of treatment with 5 μM 9-*cis*-retinal.

Compound library	Activity score range (%)	Number of compounds	Cumulative number of compounds
25K University of Cincinnati Diversity Set	50-100	1	1
	30 to 50	2	3
	20 to 30	10	**13**
	10 to 20	49	59
	<10	26,060	26,120
50K Life Chemicals Diversity Set	>100	19	19
	50-100	24	43
	30-50	31	74
	20-30	58	**132**
	10-20	314	446
	<10	50,114	50,560
2.4K Spectrum Collection	>100	1	1
	50-100	3	4
	30-50	2	6
	20-30	6	**12**
	10-20	3	15
	<10	2385	2,400
Summary of all compounds tested	>100	20	20
	50-100	28	48
	30-50	35	83
	20-30	74	**157**
	10-20	366	523
	<10	78,550	79,080

Note: The cutoff score for selection of hit compounds was 20%. Bold numbers denote the total hit number identified by the primary HTS from a total of 79,080 compounds in three small molecule libraries.

Supplementary Table 2: Summary of quality control parameters for the HTS. *S*/*B* ratio=$Mean_{100\%}/Mean_{0\%}$; *Z'*-factor = 1 – 3 X ($SD_{0\%} + SD_{100\%}$) / ($Mean_{100\%} - Mean_{0\%}$). A total of 79,080 compounds was tested in 248 assay plates. In each assay plate, $Mean_{0\%}$ and $SD_{0\%}$ provide averages and standard deviations of readouts from sixteen wells treated with DMSO, whereas $Mean_{100\%}$ and $SD_{100\%}$ are averages and standard deviations of readouts from sixteen wells, each treated with 5 μM 9-*cis*-retinal.

Library	*S*/*B* ratio		*Z'*-factor	
	Mean±s.d.	Range	Mean±s.d.	Range
25K University of Cincinnati Diversity Set	12.46±1.62	9.61—16.97	0.65±0.06	0.50—0.76
50K Life Chemicals Diversity Set	15.32±3.13	9.30—25.27	0.73±0.06	0.55—0.84
2.4K Spectrum Collection	15.71±1.85	12.64—18.24	0.75±0.03	0.71—0.80

Supplementary Table 3: Summary of The Bacteria Reverse Mutation Test in *Salmonella typhimurium* and *Escherichia coli* in 24-well plates (Micro Ames Test).

Concentration of YC-001	Average revertant colonies per well									
	TA98		TA100		TA1535		TA97a		WP2	
Without S9 Activation										
DMSO	1.3		8.4		1		9.1		28.8	
0.075	0.5		6.5		1		11.5		20.5	
0.25	1		5		3		7		19.5	
0.75	2		10		3.5		13.5		20	
2.5	2.5		12.5		1		8.5		22	
7.5	2		14		1		13.5		25	
25	3.5		0	T	0		3.5	T	25	
75 [ppt]	0		-	IL, T	-	IL, T	-	IL, T	14	T
250 [ppt]	0		-	IL, T	-	IL, T	-	IL, T	10.5	T
	23.0	+	>50.0[b]	+	>50.0[b]	+	>39.5[c]	+	>80.0[d]	+
With S9 Activation										
DMSO	2.4		10.3		0.8		11.3		34.1	
0.075	1		5.5		0.5		12.5		24	
0.25	0		5.5		1		10.5		24	
0.75	3.5		4.5		0		14.5		25	
2.5	3		6		1		11		35	
7.5	3.5		7		2		19.5		25	
25	2.5		7		1		10.5		27	
75 [ppt]	0		-	IL,T	-	NL, T	-	IL, T	17.5	T
250 [ppt]	0		-	NL,T	-	NL, T	-	IL, T	13	T
Positive control	>50.0[e]	+	>50.0[e]	+	19.0[e]	+	>50.0[e]	+	>80.0[f]	+

TA98, *S. typhimurium* TA98 *his*D3052 *rfa ΔuvrB* pKM101; TA100, *S. typhimurium* TA100 *his*G46 *rfa ΔuvrB* pKM101; TA1535, *S. typhimurium* TA1535 *his*G46 *rfa ΔuvrB;* TA97a, *S. typhimurium* TA97a *hisO1242 rfa ΔuvrB* pKM101; WP2, *E. coli* WP2 *trp uvrA* pKM101;[ppt], precipitate or insoluble material; IL, incomplete background lawn; NL, no background lawn; T, toxic as indicated by substantial concentration-related reduction in revertant colony counts, or IL/NL; +, substantial increase indicative of genotoxicity: either a response ≥ 2 times the concurrent negative control values for TA100, TA97a and WP2, or ≥ 3 times for TA98 and TA1535, with an average of at least 6 colonies; [a], 2-nitrofluorene, 0.2 μg/well; [b], sodium azide, 0.05 μg/well; [c], 9-aminoacridine hemihydrate, 2.0 μg/well; [d], 4-nitroquinoline N-oxide, 0.1 μg/well; [e], 2-aminoanthracene, 0.1 μg/well; [f], 2-aminoanthracene, 2.0 μg/well.

Supplementary Note

Compound Synthesis for Medicinal Chemistry Study of YC-001.

To understand the pharmacology of YC-001 and improve its efficacy and potency, we tested compounds related to YC-001, one modification at a time (**Fig. 1c**). These YC-001-related compounds were denoted as YC-002 to YC-063. Compounds YC-001 to YC-021 were purchased from Enamine LLC (Monmouth Jct., NJ, USA), Matrix Scientific (Columbia, SC, USA) and Tokyo Chemical Industry Co., Ltd. (Portland, OR, USA). YC-022 to YC-063 were synthesized by Charles River, Inc. (Wilmington, MA, USA). The synthesis of YC analogs was undertaken in five areas (**Fig. 1c** and **Supplementary Data 2-5**): 1) substituents at the C-3 position (moiety I); 2) substituents at the C-4 position (moiety III); 3) replacement of the furan-2(5H)-one (moiety II) with either a 1,5-dihydro-2H-pyrrol-2-one ring, a maleimide ring, or an isoxazol-5(2H)-one ring. Purity of each synthesized YC compound was analyzed by LC-MS and NMR (**Supplementary Data 6**). To synthesize YC compounds with substitutions at the C-3 or C4 positions (YC-022 to YC-57 and YC-068, **Supplementary Data 2 and 3**), reactions were undertaken following a synthetic route similar to that for YC-001, as shown in **Supplementary Fig. 9a.** Analogs of YC-001 were prepared by the condensation of a-halogeno ketones (**4**) with substituted acetic acids (**5**) followed by intramolecular cyclization of the acetate intermediate with DBU.

Replacement of the furan-2(5H)-one scaffold of YC-001(moiety II in **Fig. 1**) with a 1,5-dihydro-2H-pyrrol-2-one moiety was accomplished. Six unsaturated pyrrolidinones (**8**) (YC-058 to YC-060, YC-062, YC-063 and YC-066, **Supplementary Data 4**) were prepared according to the procedure outlined in **Supplementary Fig. 9b**. Target compounds were prepared by treatment of YC-001 with the appropriate amine followed by intramolecular cyclization of the amide intermediate (**7**) with PBr_3.

Synthesis of unsaturated pyrrolidinones was also attempted from YC-043 which has a 2-pyridyl group at the C-3 position (**Supplementary Fig. 9c**). Oxidation of the N-methyl analogue (**9**) was observed during the purification process and full conversion of **9** to the corresponding maleimide analog YC-061 was obtained when the compound was stored in solution. None of the analogs of type **8** (**Supplementary Fig. 9b**) that possess a 2-thienyl group at C-3, showed signs of oxidation.

Replacement of the furan-2(5H)-one scaffold of YC-001(moiety II in **Figure 1c**) with an isoxazol-5(2H)-one moiety also was undertaken. YC-064 was prepared according to the procedure outlined in **Supplementary Fig. 9d**. The first step of this synthesis was a Claisen condensation of ethyl 2-(thiophen-2-yl)acetate (**11**) and (5-chlorothiophen-2-yl)imidazolide (**10**) to produce the -ketoester (**12**) with sodium hydride. Cyclocondensation of hydroxylamine hydrochloride with -ketoester (**12**) then provided the core isoxazolone ring system, YC-064.

Three derivatives of type **13** YC compounds (YC-065, YC-067 and YC-069, **Supplementary Data 4**) were prepared by N-alkylation of YC-064 with triethylamine and the appropriate alkyl halide (**Supplementary Fig. 9e**).
