# Supplementary material for: A novel small molecule chaperone of rod opsin and its potential therapy for retinal degeneration
Source: Nat Commun. 2018 May 17;9:1976. doi: 10.1038/s41467-018-04261-1 (PMC5958115; doi:10.1038/s41467-018-04261-1)
Supplement: Supplementary file 2 — Description of Additional Supplementary Information [file 41467_2018_4261_MOESM2_ESM.docx]

**Description of Additional Supplementary Files**

File Name: Supplementary Data 1

Description:

**Compounds with top activities for rescuing P23H opsin transport compared to 9-*cis*-retinal.**

Note: ^a^, HCS, high-content screening; ^b^, MEM-total, the ratio of the fluorescence intensity of P23H rod opsin on the plasma membrane to that in the whole cell; ^c^, n = no; y = yes

File Name: Supplementary Data 2

Description:

**Medicinal Chemistry of YC-001 with modifications linked to the C_3_ of the furan-2(5H)-one ring ().** Activities of compounds were tested with the β-Gal fragment complementation assay to quantify the rescue of P23H opsin from the ER to the plasma membrane. Activity scores were normalized to the effect of treatment with 5 µM 9-*cis*-retinal. Compounds with efficacies greater than 20% are listed in bold type.

File Name: Supplementary Data 3

Description:

**Medicinal Chemistry of YC-001 with modifications linked to C_4_ of the furan-2(5H)-one ring ()**. Activities of compounds were tested with the β-Gal fragment complementation assay to quantify the rescue of P23H opsin from the ER to the plasma membrane. Activity scores are normalized to the effect of treatment with 5 µM 9-*cis*-retinal. Compounds with efficacies higher than 20% are listed in bold type.

File Name: Supplementary Data 4

Description:

**Medicinal Chemistry of YC-001 with modifications of the furan-2(5H)-one ring.** Activities of the compounds were tested with the β-Gal fragment complementation assay to quantify the rescue of P23H opsin from the ER to the plasma membrane. Activity scores were normalized to the effect of treatment with 5 µM 9-*cis*-retinal. Only YC-001 showed an efficacy higher than 20% and is listed in bold type.

File Name: Supplementary Data 5

Description:

**Medicinal chemistry of YC-001 with more than one site modified.** Activities of compounds were tested with the β-Gal fragment complementation assay to quantify the rescue of P23H opsin from the ER to the plasma membrane. Activity scores are normalized to the effect of treatment with 5 µM 9-*cis*-retinal. Only YC-001 showed an efficacy higher than 20% and is listed in bold type

File Name: Supplementary Data 6

Description:

**LC-MS and NMR data for YC-001 and YC-022-069 synthesized by Charles River, Inc.**

Note:

Method 1: UPLC-MS was performed on a UPLC Acquity with AcquityPDA Detector coupled to a Micromass ZQ, Acquity QDA detector single quadrupole mass spectrometer using a Waters BEH column C18 (1.7 µm, 50 x 2.1mm) with a linear gradient of 1-99.9 % acetonitrile/water (with 0.05% formic acid in each mobile phase) within 4 minutes and held at 99.9% for 3.5 minutes; F=1 ml/min.

Method 2: UPLC-MS was performed on a Waters Acquity I-Class with Waters Diode Array Detector coupled to a Waters SQD2 single quadrupole mass spectrometer using an Waters BEH Shield RP18 column (1.7 µm, 100 × 2.1 mm) being initially held at 5% acetonitrile/water (with 10 mM ammonium bicarbonate in each mobile phase) for 1.2 minutes, followed by a linear gradient of 5-100% within 3.5 minutes and then held at 100% for 1.5 minutes (F = 0.5 mL/min).

*SMILES: the simplified molecular-input line-entry system.
