# Supplementary material for: A novel small molecule chaperone of rod opsin and its potential therapy for retinal degeneration
Source: Nat Commun. 2018 May 17;9:1976. doi: 10.1038/s41467-018-04261-1 (PMC5958115; doi:10.1038/s41467-018-04261-1)
Supplement: Supplementary file 3 — Supplementary Data 1 [file 41467_2018_4261_MOESM3_ESM.docx]

**Supplementary Data 1: Compounds with top activities for rescuing P23H opsin transport compared to 9-*cis*-retinal.**

| Compound name | Structure | β-Gal fragment complementation assay  Activity score (%) | | | Immunostaining HCS^a^  MEM-Total ratio^b^ | | Improved glycosylation^c^ |
| --- | --- | --- | --- | --- | --- | --- | --- |
|  |  | EC_50_ (µM) | Max (fit) | Max (Exp) | EC_50_ (µM) | Max (fit) |  |
| Scriptaid |  | 3.09 | 98.2 | 98 | 0.38 | 0.070 | n |
| F5581-0240 |  | 5.93 | 230 | 244 | 0.64 | 0.010 | y |
| F6257-1860 |  | 10.7 | 234 | 211 | 1.07 | 0.022 | n |
| F5937-0207 |  | 8.38 | 37.3 | 32.8 | 1.33 | 0.021 | n |
| F3382-5924 |  | 28.6 | 155 | 931 | 2.42 | 0.046 | y |
| **YC-001** |  | **8.7** | **310** | **310** | **2.75** | **0.030** | **y** |
| F2902-1134 |  | 21.6 | 31.5 | 23.1 | 4.15 | 0.031 | n |
| F6244-1415 |  | 8.61 | 25.2 | 24.8 | 4.2 | 0.063 | n |
| F5875-0200 |  | 25.0 | 51.6 | 31.8 | 6.2 | 0.041 | n |
| F5111-0031 |  | 54.3 | 165 | 60.4 | 6.76 | 0.025 | y |
| 9-*cis*-retinal |  | 2.7 | 159 | 123 | 0.377 | 0.019 | y |

Note: ^a^, HCS, high-content screening; ^b^, MEM-total, the ratio of the fluorescence intensity of P23H rod opsin on the plasma membrane to that in the whole cell; ^c^, n = no; y = yes
