# Supplementary material for: A novel small molecule chaperone of rod opsin and its potential therapy for retinal degeneration
Source: Nat Commun. 2018 May 17;9:1976. doi: 10.1038/s41467-018-04261-1 (PMC5958115; doi:10.1038/s41467-018-04261-1)
Supplement: Supplementary file 4 — Supplementary Data 2 [file 41467_2018_4261_MOESM4_ESM.docx]

**Supplementary** **Data 2:** **Medicinal Chemistry of YC-001 with modifications linked to the C_3_ of the furan-2(5H)-one ring ().** Activities of compounds were tested with the β-Gal fragment complementation assay to quantify the rescue of P23H opsin from the ER to the plasma membrane. Activity scores were normalized to the effect of treatment with 5 µM 9-*cis*-retinal. Compounds with efficacies greater than 20% are listed in bold type.

| Number | Compound name | R | Molecular weight | Potency  EC_50_ (µM) | Efficacy (%) |
| --- | --- | --- | --- | --- | --- |
| 1 | **YC-001** | 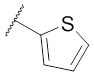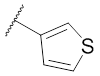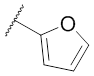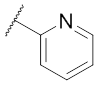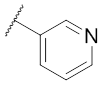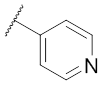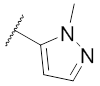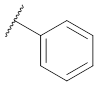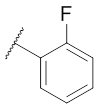 | 282.77 | 8.39 | 248 |
| 2 | **YC-051** |  | 282.77 | 10.90 | 169 |
| 3 | YC-028 |  | 266.70 | NA | NA |
| 4 | **YC-043** |  | 277.73 | 19.00 | 214 |
| 5 | YC-030 |  | 277.73 | NA | NA |
| 6 | YC-031 |  | 277.73 | NA | NA |
| 7 | YC-057 |  | 280.73 | NA | NA |
| 8 | **YC-047** |  | 276.74 | 9.87 | 188 |
| 9 | **YC-068** |  | 294.73 | 5.66 | 261 |
| 10 | **YC-032** | 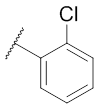 | 311.18 | 5.80 | 288 |
| 11 | **YC-013** | 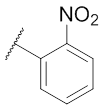 | 321.73 | 11.0 | 111 |
| 12 | **YC-033** | 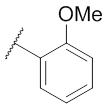 | 306.76 | 8.50 | 38.3 |
| 13 | YC-036 | 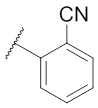 | 301.75 | NA | NA |
| 14 | **YC-050** | 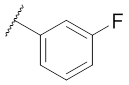 | 294.73 | 7.36 | 131 |
| 15 | **YC-034** | 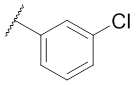 | 311.18 | 4.75 | 43.5 |
| 16 | YC-041 | 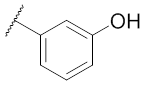 | 292.74 | NA | NA |
| 17 | YC-045 | 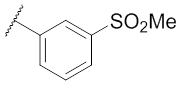 | 354.83 | NA | NA |
| 19 | **YC-053** | 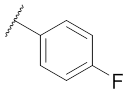 | 294.73 | 24.1 | 32.9 |
| 20 | YC-027 | 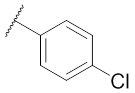 | 311.18 | NA | NA |
| 21 | YC-023 | 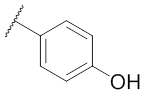 | 292.74 | NA | NA |
| 22 | YC-052 | 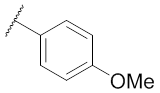 | 306.77 | 7.13 | 10 |
| 23 | YC-014 | 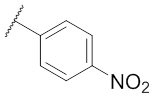 | 321.73 | NA | NA |
| 24 | YC-039 | 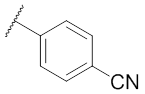 | 301.75 | NA | NA |
| 25 | YC-022 | 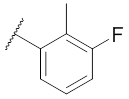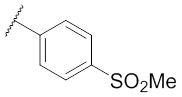 | 354.83 | NA | NA |
| 26 | **YC-054** |  | 308.76 | 3.53 | 227 |
| 27 | **YC-056** | 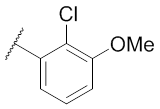 | 341.21 | 9.39 | 104 |
| 28 | YC-055 | 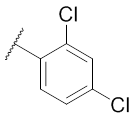 | 345.63 | 5.33 | 11 |
