# Supplementary material for: A novel small molecule chaperone of rod opsin and its potential therapy for retinal degeneration
Source: Nat Commun. 2018 May 17;9:1976. doi: 10.1038/s41467-018-04261-1 (PMC5958115; doi:10.1038/s41467-018-04261-1)
Supplement: Supplementary file 5 — Supplementary Data 3 [file 41467_2018_4261_MOESM5_ESM.docx]

**Supplementary** **Data 3:** **Medicinal Chemistry of YC-001 with modifications linked to C_4_ of the furan-2(5H)-one ring ()**. Activities of compounds were tested with the β-Gal fragment complementation assay to quantify the rescue of P23H opsin from the ER to the plasma membrane. Activity scores are normalized to the effect of treatment with 5 µM 9-*cis*-retinal. Compounds with efficacies higher than 20% are listed in bold type.

| Number | Compound name | R | Molecular weight | Potency (µM) | Efficacy (%) |
| --- | --- | --- | --- | --- | --- |
| 1 | **YC-001** | 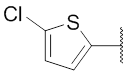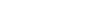 | 282.77 | 8.39 | 248 |
| 2 | **YC-021** | 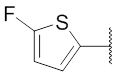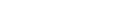 | 266.30 | 103.00 | 160 |
| 3 | YC-002 | 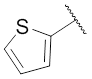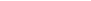 | 248.31 | 33.00 | 6 |
| 4 | **YC-049** | 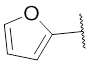 | 232.26 | 91.90 | 112 |
| 5 | YC-026 | 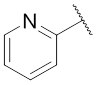 | 243.28 | NA | NA |
| 6 | YC-046 | 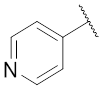 | 243.28 | NA | NA |
| 7 | YC-025 | 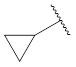 | 206.26 | NA | NA |
| 8 | YC-024 | 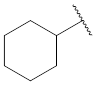 | 248.34 | NA | NA |
| 9 | YC-038 | 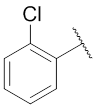 | 276.74 | NA | NA |
| 10 | YC-042 | 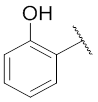 | 258.29 | NA | NA |
| 11 | YC-035 | 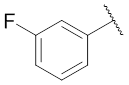 | 260.28 | NA | NA |
| 12 | YC-048 | 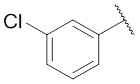 | 276.74 | NA | NA |
| 13 | YC-040 | 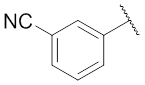 | 267.30 | NA | NA |
| 14 | YC-044 | 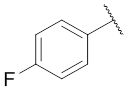 | 260.28 | 37.60 | 10 |
| 15 | YC-003 | 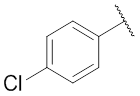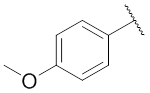 | 276.74 | 22.00 | 5 |
| 16 | YC-037 |  | 272.32 | NA | NA |
