# Supplementary material for: A novel small molecule chaperone of rod opsin and its potential therapy for retinal degeneration
Source: Nat Commun. 2018 May 17;9:1976. doi: 10.1038/s41467-018-04261-1 (PMC5958115; doi:10.1038/s41467-018-04261-1)
Supplement: Supplementary file 6 — Supplementary Data 4 [file 41467_2018_4261_MOESM6_ESM.docx]

**Supplementary** **Data 4:** **Medicinal Chemistry of YC-001 with modifications of the furan-2(5H)-one ring.** Activities of the compounds were tested with the β-Gal fragment complementation assay to quantify the rescue of P23H opsin from the ER to the plasma membrane. Activity scores were normalized to the effect of treatment with 5 µM 9-*cis*-retinal. Only YC-001 showed an efficacy higher than 20% and is listed in bold type.

| Number | Compound name | Scaffold | R | Molecular weight | Potency (µM) | Efficacy (%) |
| --- | --- | --- | --- | --- | --- | --- |
| 1 | **YC-001** | 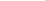  |  | 282.77 | 8.39 | 248 |
| 2 | YC-060 |  |  | 281.78 | 24.6 | 7.1 |
| 3 | YC-058 |  |  | 295.81 | NA | NA |
| 4 | YC-063 |  | 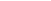  | 323.864 | NA | NA |
| 5 | YC-059 |  | 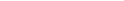  | 339.86 | NA | NA |
| 6 | YC-062 |  |  | 353.89 | NA | NA |
| 7 | YC-066 |  | 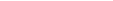  | 379.928 | NA | NA |
| 8 | YC-064 |  | 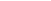  | 283.755 | NA | NA |
| 9 | YC-065 |  | 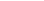  | 297.782 | NA | NA |
| 10 | YC-067 |  |  | 373.88 | NA | NA |
| 11 | YC-069 |  |  | 337.84 | NA | NA |
| 12 | YC-061 |  |  | 304.75 | NA | NA |
