# Supplementary material for: A novel small molecule chaperone of rod opsin and its potential therapy for retinal degeneration
Source: Nat Commun. 2018 May 17;9:1976. doi: 10.1038/s41467-018-04261-1 (PMC5958115; doi:10.1038/s41467-018-04261-1)
Supplement: Supplementary file 7 — Supplementary Data 5 [file 41467_2018_4261_MOESM7_ESM.docx]

**Supplementary** **Data 5:** **Medicinal chemistry of YC-001 with more than one site modified.** Activities of compounds were tested with the β-Gal fragment complementation assay to quantify the rescue of P23H opsin from the ER to the plasma membrane. Activity scores are normalized to the effect of treatment with 5 µM 9-*cis*-retinal. Only YC-001 showed an efficacy higher than 20% and is listed in bold type.

| Number | Compound name | Scaffold | Rs | Molecular weight | Potency (µM) | Efficacy (%) |
| --- | --- | --- | --- | --- | --- | --- |
| 1 | **YC-001** | 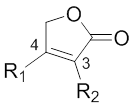 | 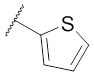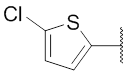 | 282.77 | 8.39 | 248 |
| 2 | YC-009 | 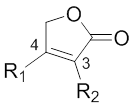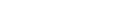 | 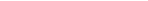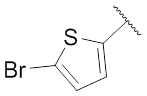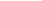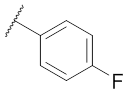 | 339.18 | 7.20 | 10 |
| 3 | YC-011 | 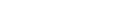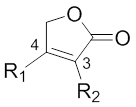 | 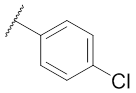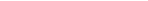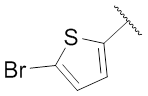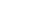 | 355.63 | 7.50 | 10 |
| 4 | YC-008 | 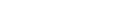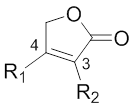 | 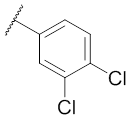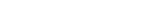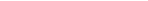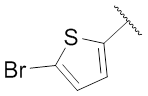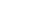 | 390.07 | 25.00 | 11 |
| 5 | YC-016 | 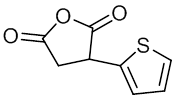 |  | 182.19 | NA | NA |
| 6 | **YC-018** | 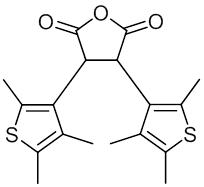 |  | 348.48 | 138.00 | 95 |
