# Supplementary material for: A novel small molecule chaperone of rod opsin and its potential therapy for retinal degeneration
Source: Nat Commun. 2018 May 17;9:1976. doi: 10.1038/s41467-018-04261-1 (PMC5958115; doi:10.1038/s41467-018-04261-1)
Supplement: Supplementary file 8 — Supplementary Data 6 [file 41467_2018_4261_MOESM8_ESM.docx]

**Supplementary Data 6: LC-MS and NMR data for YC-001 and YC-022-069 synthesized by Charles River, Inc.**

| Compound Name | LC-MS | NMR | Formula | Molecular weight | Purity (%) | SMILES_STRUCTURE* |
| --- | --- | --- | --- | --- | --- | --- |
| YC-001 | LCMS (Method 1): [MH+] = 283 at 3.58 min | ¹H NMR (400 MHz, CD3CN): δ 7.70 (dd, J = 1.2, 5.1 Hz, 1 H), 7.39 (dd, J = 1.2, 3.6 Hz, 1 H), 7.31 (d, J = 4.1 Hz, 1 H), 7.24 (dd, J = 3.6, 5.1 Hz, 1 H), 7.08 (d, J = 4.1 Hz, 1 H), 5.26 (s, 2 H). | C12H7ClO2S2 | 282.77 | 95.87 | C1\C(=C(/C(O1)=O)\c1cccs1)\c1sc(cc1)Cl |
| YC-022 | LCMS (Method 1): [MH+] = 355 at 3.17 min | ¹H NMR (400 MHz, CD3CN): δ 8.08 (d, J = 8.5 Hz, 2 H), 7.71 (d, J = 8.5 Hz, 2 H), 7.27 (d, J = 4.1 Hz, 1 H), 7.07 (d, J = 4.0 Hz, 1 H), 5.30 (s, 2 H), 3.17 (s, 3 H). | C15H11ClO4S2 | 354.82843 | 98.68 | Clc1sc(C=2COC(=O)C2c2ccc(cc2)S(=O)(=O)C)cc1 |
| YC-023 | LCMS (Method 1): [MH+] = 293 at 3.18 min | ¹H NMR (400 MHz, CD3CN): δ 7.38 (s, 1 H), 7.30-7.26 (m, 2 H), 7.22 (d, J = 3.8 Hz, 1 H), 7.03 (d, J = 4.2 Hz, 1 H), 7.00-6.95 (m, 2 H), 5.24 (s, 2 H). | C14H9ClO3S | 292.73745 | 97.61 | Oc1ccc(C2=C(COC2=O)c2sc(Cl)cc2)cc1 |
| YC-024 | LCMS (Method 1): [MH+] = 249 at 3.73 min | ¹H NMR (400 MHz, CD3CN): δ 7.61 (d, J = 3.5 Hz, 1 H), 7.56 (dd, J = 1.0, 5.3 Hz, 1 H), 7.19 (dd, J = 3.8, 5.1 Hz, 1 H), 4.92 (s, 2 H), 3.19-3.12 (m, 1 H), 1.91-1.74 (m, 5 H), 1.47-1.38 (m, 4 H), 1.36-1.27 (m, 1 H). | C14H16O2S | 248.34064 | 92.71 | O=C1C(c2sccc2)=C(CO1)C1CCCCC1 |
| YC-025 | LCMS (Method 1): [MH+] = 207 at 3.22 min | ¹H NMR (400 MHz, CD3CN): δ 7.71 (d, J = 3.6 Hz, 1 H), 7.56 (d, J = 5.0 Hz, 1 H), 7.20 (dd, J = 3.6, 5.1 Hz, 1 H), 4.65 (s, 2 H), 2.40-2.33 (m, 1 H), 1.23-1.17 (m, 2 H), 0.96-0.92 (m, 2 H). | C11H10O2S | 206.2609 | 94.93 | O=C1OCC(=C1c1cccs1)C1CC1 |
| YC-026 | LCMS (Method 2): [MH+] = 244 at 3.06 min | ¹H NMR (400 MHz, CD3CN): δ 8.72 (d, J = 4.3 Hz, 1 H), 7.83-7.78 (m, 1 H), 7.66 (d, J = 8.1 Hz, 1 H), 7.60-7.55 (m, 2 H), 7.44 (ddd, J = 1.3, 4.8, 7.6 Hz, 1 H), 7.15 (dd, J = 3.7, 5.2 Hz, 1 H), 5.31 (s, 2 H). | C13H9NO2S | 243.28105 | 92.32 | O=C1C(c2cccs2)=C(c2ncccc2)CO1 |
| YC-027 | LCMS (Method 1): [MH+] = 311 at 3.75 min | ¹H NMR (400 MHz, CD3CN): δ 7.58-7.55 (m, 2 H), 7.46-7.43 (m, 2 H), 7.24 (d, J = 4.0 Hz, 1 H), 7.05 (d, J = 4.0 Hz, 1 H), 5.27 (s, 2 H). | C14H8Cl2O2S | 311.18311 | 96.89 | O=C1OCC(=C1c1ccc(cc1)Cl)c1sc(Cl)cc1 |
| YC-028 | LCMS (Method 1): [MH+] = 267 at 3.56 min | ¹H NMR (400 MHz, CD3CN): δ 7.76 (d, J = 1.8 Hz, 1 H), 7.42 (d, J = 4.1 Hz, 1 H), 7.17 (d, J = 3.5 Hz, 1 H), 7.12 (d, J = 4.1 Hz, 1 H), 6.66 (dd, J = 1.8, 3.5 Hz, 1 H), 5.22 (s, 2 H). | C12H7ClO3S | 266.70017 | 97.94 | O=C1C(=C(c2sc(cc2)Cl)CO1)c1ccco1 |
| YC-030 | LCMS (Method 1): [MH+] = 278 at 2.95 min | ¹H NMR (400 MHz, CD3CN) d 8.72 (dd, J=1.7, 4.8 Hz, 1H), 8.64 (d, J=1.9 Hz, 1H), 7.85 - 7.82 (m, 1H), 7.53 (dd, J=4.9, 7.5 Hz, 1H), 7.27 (d, J=4.1 Hz, 1H), 7.07 (d, J=4.1 Hz, 1H), 5.31 (s, 2H). | C12H7ClO2S2 | 282.76577 | 97.08 | O=C1OCC(c2sc(cc2)Cl)=C1c1cccs1 |
| YC-031 | LCMS (Method 1): [MH+] = 278 at 2.75 min | ¹H NMR (400 MHz, CD3CN): δ 8.75-8.73 (m, 2 H), 7.44-7.42 (m, 2 H), 7.26 (d, J = 4.1 Hz, 1 H), 7.06 (d, J = 4.0 Hz, 1 H), 5.28 (s, 2 H). | C13H8ClNO2S | 277.72611 | 98.91 | Clc1ccc(C2=C(c3cccnc3)C(=O)OC2)s1 |
| YC-032 | LCMS (Method 1): [MH+] = 311 at 3.64 min | ¹H NMR (400 MHz, CD3CN): δ 7.64 (dd, J = 1.1, 8.0 Hz, 1 H), 7.60-7.49 (m, 2 H), 7.40 (dd, J = 1.4, 7.5 Hz, 1 H), 7.24 (d, J = 4.0 Hz, 1 H), 7.05 (d, J = 4.1 Hz, 1 H), 5.36 (dd, J = 16.6, 27.2 Hz, 2 H). | C13H8ClNO2S | 277.72611 | 95.03 | Clc1sc(C2=C(C(OC2)=O)c2ccncc2)cc1 |
| YC-033 | LCMS (Method 1): [MH+] = 307 at 3.53 min | ¹H NMR (400 MHz, CD3CN): δ 7.57-7.52 (m, 1 H), 7.25-7.19 (m, 2 H), 7.17-7.09 (m, 2 H), 7.02 (d, J = 4.0 Hz, 1 H), 5.30 (s, 2 H), 3.75 (s, 3 H). | C14H8Cl2O2S | 311.18311 | 99.56 | Clc1ccc(C2=C(c3ccccc3Cl)C(OC2)=O)s1 |
| YC-034 | LCMS (Method 1): [MH+] = 311 at 3.73 min | ¹H NMR (400 MHz, CD3CN): δ 7.57-7.54 (m, 2 H), 7.47 (d, J = 1.9 Hz, 1 H), 7.41-7.38 (m, 1 H), 7.25 (d, J = 4.1 Hz, 1 H), 7.06 (d, J = 4.0 Hz, 1 H), 5.28 (s, 2 H). | C15H11ClO3S | 306.76403 | 98.88 | O=C1OCC(c2sc(cc2)Cl)=C1c1ccccc1OC |
| YC-035 | LCMS (Method 1): [MH+] = 261 at 3.44 min | ¹H NMR (400 MHz, CD3CN): δ 7.57-7.50 (m, 2 H), 7.47 (d, J = 3.6 Hz, 1 H), 7.35-7.24 (m, 3 H), 7.10 (dd, J = 3.7, 5.1 Hz, 1 H), 5.14 (s, 2 H). | C14H8Cl2O2S | 311.18311 | 97.31 | O=C1C(c2cccc(c2)Cl)=C(c2sc(cc2)Cl)CO1 |
| YC-036 | LCMS (Method 1): [MH+] = 302 at 3.34 min | ¹H NMR (400 MHz, CD3CN): δ 7.95 (d, J = 7.8 Hz, 1 H), 7.88-7.83 (m, 1 H), 7.75-7.70 (m, 1 H), 7.58 (d, J = 7.8 Hz, 1 H), 7.28 (d, J = 4.1 Hz, 1 H), 7.07 (d, J = 4.1 Hz, 1 H), 5.40 (dd, J = 15.9, 38.0 Hz, 2 H). | C14H9FO2S | 260.28346 | 94.95 | O=C1C(=C(c2cc(F)ccc2)CO1)c1cccs1 |
| YC-037 | LCMS (Method 1): [MH+] = 273 at 3.5 min | ¹H NMR (400 MHz, CD3CN): δ 7.51-7.45 (m, 3 H), 7.42 (d, J = 3.0 Hz, 1 H), 7.11 (dd, J = 3.6, 5.1 Hz, 1 H), 7.03-7.00 (m, 2 H), 5.15 (s, 2 H), 3.86 (s, 3 H). | C15H8ClNO2S | 301.74751 | 96.89 | O=C1OCC(c2ccc(Cl)s2)=C1c1c(cccc1)C#N |
| YC-038 | LCMS (Method 1): [MH+] = 277 at 3.58 min | ¹H NMR (400 MHz, CD3CN): δ 7.56-7.43 (m, 6 H), 7.10 (dd, J = 3.8, 5.1 Hz, 1 H), 5.14 (s, 2 H). | C15H12O3S | 272.31897 | 96.24 | COc1ccc(cc1)C1=C(C(OC1)=O)c1cccs1 |
| YC-039 | LCMS (Method 1): [MH+] = 302 at 3.42 min | ¹H NMR (400 MHz, CD3CN): δ 7.89 (d, J = 8.4 Hz, 2 H), 7.63 (d, J = 8.4 Hz, 2 H), 7.26 (d, J = 3.8 Hz, 1 H), 7.07 (d, J = 4.2 Hz, 1 H), 5.29 (s, 2 H). | C14H9ClO2S | 276.73805 | 96.7 | Clc1ccccc1C=1COC(=O)C1c1cccs1 |
| YC-040 | LCMS (Method 1): [MH+] = 268 at 3.26 min | ¹H NMR (400 MHz, CD3CN): δ 7.87 (d, J = 7.8 Hz, 2 H), 7.79-7.77 (m, 1 H), 7.67 (dd, J = 7.7, 7.7 Hz, 1 H), 7.51 (d, J = 5.0 Hz, 1 H), 7.46 (d, J = 3.4 Hz, 1 H), 7.10 (dd, J = 3.7, 5.1 Hz, 1 H), 5.14 (s, 2 H). | C15H8ClNO2S | 301.74751 | 96.8 | Clc1sc(C2=C(c3ccc(C#N)cc3)C(=O)OC2)cc1 |
| YC-041 | LCMS (Method 1): [MH+] = 293 at 3.2 min | ¹H NMR (400 MHz, CD3CN): δ 7.37 (dd, J = 8.0, 8.0 Hz, 1 H), 7.22 (d, J = 4.0 Hz, 1 H), 7.18 (brs, 1 H), 7.03 (d, J = 4.0 Hz, 1 H), 6.98-6.96 (m, 1 H), 6.91 (d, J = 7.5 Hz, 1 H), 6.86-6.84 (m, 1 H), 5.24 (s, 2 H). | C15H9NO2S | 267.30246 | 93.37 | O=C1C(=C(c2cccc(C#N)c2)CO1)c1cccs1 |
| YC-042 | LCMS (Method 1): [MH+] = 259 at 3.17 min | ¹H NMR (400 MHz, CD3CN): δ 7.50 (d, J = 3.6 Hz, 1 H), 7.44-7.36 (m, 2 H), 7.29-7.23 (m, 2 H), 7.06-7.00 (m, 3 H), 5.12 (s, 2 H). | C14H9ClO3S | 292.73745 | 96.45 | Clc1sc(C2=C(C(OC2)=O)c2cccc(c2)O)cc1 |
| YC-043 | LCMS (Method 1): [MH+] = 278 at 3.43 min | ¹H NMR (400 MHz, CD3CN): δ 8.80 (d, J = 4.8 Hz, 1 H), 8.10 (d, J = 8.1 Hz, 1 H), 7.97-7.91 (m, 1 H), 7.49-7.42 (m, 2 H), 7.12 (d, J = 4.3 Hz, 1 H), 5.35 (s, 2 H). | C14H10O3S | 258.2924 | 98.25 | Oc1ccccc1C1=C(c2sccc2)C(=O)OC1 |
| YC-044 | LCMS (Method 1): [MH+] = 261 at 3.44 min | ¹H NMR (400 MHz, CD3CN): δ 7.56-7.49 (m, 3 H), 7.45 (d, J = 3.5 Hz, 1 H), 7.25 (dd, J = 8.9, 8.9 Hz, 2 H), 7.09 (dd, J = 3.7, 5.1 Hz, 1 H), 5.14 (s, 2 H). | C13H8ClNO2S | 277.72611 | 97.51 | Clc1ccc(C2=C(c3ccccn3)C(=O)OC2)s1 |
| YC-045 | LCMS (Method 1): [MH+] = 355 at 3.18 min | ¹H NMR (400 MHz, CD3CN): δ 8.09-8.05 (m, 1 H), 8.00 (s, 1 H), 7.80-7.78 (m, 2 H), 7.26 (d, J = 4.0 Hz, 1 H), 7.06 (d, J = 4.3 Hz, 1 H), 5.29 (s, 2 H), 3.14 (s, 3 H). | C14H9FO2S | 260.28346 | 97.73 | Fc1ccc(cc1)C1=C(C(OC1)=O)c1sccc1 |
| YC-046 | LCMS (Method 1): [MH+] = 244 at 2.72 min | ¹H NMR (400 MHz, CD3CN): δ 8.81-8.78 (m, 2 H), 7.80-7.77 (m, 2 H), 7.58 (dd, J = 1.1, 5.2 Hz, 1 H), 7.46-7.45 (m, 1 H), 7.11 (dd, J = 3.8, 5.3 Hz, 1 H), 5.18 (s, 2 H). | C15H11ClO4S2 | 354.83 | 98.5 | CS(c1cccc(c1)C=1C(=O)OCC1c1sc(cc1)Cl)(=O)=O |
| YC-047 | LCMS (Method 1): [MH+] = 277 at 3.6 min | ¹H NMR (400 MHz, CD3CN): δ 7.56-7.53 (m, 3 H), 7.44-7.41 (m, 2 H), 7.22 (d, J = 4.0 Hz, 1 H), 7.03 (d, J = 4.0 Hz, 1 H), 5.26 (s, 2 H). | C14H9ClO2S | 276.74 | 99.91 | C1\C(=C(/C(O1)=O)\c1ccccc1)\c1sc(cc1)Cl |
| YC-048 | LCMS (Method 1): [MH+] = 277 at 3.66 min | ¹H NMR (400 MHz, CD3CN): δ 7.56-7.43 (m, 6 H), 7.10 (dd, J = 3.7, 5.1 Hz, 1 H), 5.14 (s, 2 H). | C14H9ClO2S | 276.74 | 93.71 | C1\C(=C(/C(O1)=O)\c1cccs1)\c1cc(ccc1)Cl |
| YC-049 | LCMS (Method 1): [MH+] = 233 at 3.28 min | ¹H NMR (400 MHz, CD3CN): δ 7.79-7.77 (m, 2 H), 7.63 (d, J = 4.4 Hz, 1 H), 7.22 (dd, J = 3.7, 5.1 Hz, 1 H), 7.08 (d, J = 3.6 Hz, 1 H), 6.68 (dd, J = 1.8, 3.6 Hz, 1 H), 5.22 (s, 2 H). | C12H8O3S | 232.26 | 98.22 | C1\C(=C(/C(O1)=O)\c1cccs1)\c1occc1 |
| YC-050 | LCMS (Method 1): [MH+] = 295 at 3.56 min | ¹H NMR (400 MHz, CD3CN): δ 7.60-7.54 (m, 1 H), 7.32-7.25 (m, 2 H), 7.24 (d, J = 4.1 Hz, 1 H), 7.22-7.18 (m, 1 H), 7.05 (d, J = 4.0 Hz, 1 H), 5.27 (s, 2 H). | C14H8ClFO2S | 294.73 | 97.21 | C1\C(=C(/C(O1)=O)\c1cccc(c1)F)\c1sc(cc1)Cl |
| YC-051 | LCMS (Method 1): [MH+] = 283 at 3.52 min | ¹H NMR (400 MHz, CD3CN): δ 7.67 (dd, J = 1.3, 3.0 Hz, 1 H), 7.61 (dd, J = 2.9, 5.0 Hz, 1 H), 7.25 (d, J = 4.0 Hz, 1 H), 7.21 (dd, J = 1.3, 5.0 Hz, 1 H), 7.06 (d, J = 4.1 Hz, 1 H), 5.24 (s, 2 H). | C12H7ClO2S2 | 282.77 | 99.77 | C1\C(=C(/C(O1)=O)\c1ccsc1)\c1sc(cc1)Cl |
| YC-052 | LCMS (Method 1): [MH+] = 307 at 3.54 min | ¹H NMR (400 MHz, CD3CN): δ 7.18-7.13 (m, 2 H), 7.01 (d, J = 4.3 Hz, 1 H), 6.90-6.86 (m, 2 H), 6.83 (d, J = 4.3 Hz, 1 H), 5.04 (s, 2 H), 3.68 (s, 3 H). | C15H11ClO3S | 306.77 | 98.12 | C1\C(=C(/C(O1)=O)\c1ccc(cc1)OC)\c1sc(cc1)Cl |
| YC-053 | LCMS (Method 1): [MH+] = 295 at 3.57 min | ¹H NMR (400 MHz, CD3CN): δ 7.48-7.44 (m, 2 H), 7.29 (dd, J = 9.0, 9.0 Hz, 2 H), 7.23 (d, J = 4.0 Hz, 1 H), 7.04 (d, J = 4.0 Hz, 1 H), 5.26 (s, 2 H). | C14H8ClFO2S | 294.73 | 99.46 | C1\C(=C(/C(O1)=O)\c1ccc(cc1)F)\c1sc(cc1)Cl |
| YC-054 | LCMS (Method 1): [MH+] = 309 at 3.68 min | ¹H NMR (400 MHz, CD3CN): δ 7.41-7.35 (m, 1 H), 7.29-7.24 (m, 1 H), 7.22 (d, J = 4.0 Hz, 1 H), 7.08 (d, J = 7.6 Hz, 1 H), 7.04 (d, J = 4.0 Hz, 1 H), 5.40-5.28 (m, 2 H), 2.11 (s, 3 H). | C15H10ClFO2S | 308.76 | 99.91 | C1\C(=C(/C(O1)=O)\c1cccc(c1C)F)\c1sc(cc1)Cl |
| YC-055 | LCMS (Method 1): [MH+] = 345 at 3.84 min | ¹H NMR (400 MHz, CD3CN): δ 7.73 (d, J = 2.0 Hz, 1 H), 7.54 (dd, J = 2.0, 8.3 Hz, 1 H), 7.39 (d, J = 8.3 Hz, 1 H), 7.25 (d, J = 4.0 Hz, 1 H), 7.07 (d, J = 4.0 Hz, 1 H), 5.36 (dd, J = 17.0, 29.1 Hz, 2 H). | C14H7Cl3O2S | 345.63 | 99.33 | C1\C(=C(/C(O1)=O)\c1ccc(cc1Cl)Cl)\c1sc(cc1)Cl |
| YC-056 | LCMS (Method 1): [MH+] = 341 at 3.54 min | ¹H NMR (400 MHz, CD3CN): δ 7.47 (dd, J = 8.0, 8.0 Hz, 1 H), 7.28-7.22 (m, 2 H), 7.04 (d, J = 4.3 Hz, 1 H), 6.97 (dd, J = 1.3, 7.6 Hz, 1 H), 5.35 (dd, J = 16.0, 27.4 Hz, 2 H), 3.98 (s, 3 H). | C15H10Cl2O3S | 341.21 | 99.44 | C1\C(=C(/C(O1)=O)\c1cccc(c1Cl)OC)\c1sc(cc1)Cl |
| YC-057 | LCMS (Method 1): [MH+] = 281 at 3.09 min | ¹H NMR (400 MHz, CD3CN): δ 7.61 (d, J = 2.0 Hz, 1 H), 7.30 (d, J = 4.1 Hz, 1 H), 7.09 (d, J = 4.1 Hz, 1 H), 6.43 (d, J = 1.9 Hz, 1 H), 5.36 (s, 2 H), 3.71 (s, 3 H). | C12H9ClN2O2S | 280.73 | 91.77 | C1\C(=C(/C(O1)=O)\c1ccnn1C)\c1sc(cc1)Cl |
| YC-058 | LCMS (Method 1): [MH+] = 296 at 3.45 min | ¹H NMR (400 MHz, CD3CN): δ 7.62 (dd, J = 1.1, 5.2 Hz, 1 H), 7.35 (dd, J = 1.0, 3.5 Hz, 1 H), 7.19-7.16 (m, 2 H), 7.01 (d, J = 4.0 Hz, 1 H), 4.37 (s, 2 H), 3.06 (s, 3 H). | C13H10ClNOS2 | 295.81 | 97.19 | C1\C(=C(/C(N1C)=O)\c1cccs1)\c1sc(cc1)Cl |
| YC-059 | LCMS (Method 1): [MH+] = 340 at 3.59 min | ¹H NMR (400 MHz, CD3CN): δ 7.50 (dd, J = 1.0, 5.1 Hz, 1 H), 7.22 (dd, J = 1.0, 3.5 Hz, 1 H), 7.08-7.04 (m, 2 H), 6.88 (d, J = 4.0 Hz, 1 H), 4.34 (s, 2 H), 3.55-3.45 (m, 4 H), 3.23 (s, 3 H). | C15H14ClNO2S2 | 339.86 | 90.26 | C1\C(=C(/C(N1CCOC)=O)\c1cccs1)\c1sc(cc1)Cl |
| YC-060 | LCMS (Method 1): [MH+] = 282 at 3.34 min | ¹H NMR (400 MHz, CD3CN): δ 7.63 (d, J = 5.1 Hz, 1 H), 7.29 (d, J = 3.4 Hz, 1 H), 7.22-7.17 (m, 2 H), 7.00 (d, J = 4.0 Hz, 1 H), 4.40 (s, 2 H). NH not observed. | C12H8ClNOS2 | 281.78 | 94.68 | C1\C(=C(/C(N1)=O)\c1cccs1)\c1sc(cc1)Cl |
| YC-061 | LCMS (Method 2): [MH+] = 305 at 3.53 min | ¹H NMR (400 MHz, DMSO): δ 8.87-8.86 (m, 1 H), 8.08-8.04 (m, 2 H), 7.84 (d, J = 7.8 Hz, 1 H), 7.61 (ddd, J = 1.3, 4.8, 7.6 Hz, 1 H), 7.29 (d, J = 4.3 Hz, 1 H), 3.08 (s, 3 H). | C14H9ClN2O2S | 304.75 | 94.82 | C1(\C(=C(/C(N1C)=O)\c1ccccn1)\c1sc(cc1)Cl)=O |
| YC-062 | LCMS (Method 2): [MH+] = 354 at 3.47 min | ¹H NMR (400 MHz, CD3CN): δ 7.63 (d, J = 5.1 Hz, 1 H), 7.35 (d, J = 2.6 Hz, 1 H), 7.21-7.17 (m, 2 H), 7.02 (d, J = 4.0 Hz, 1 H), 4.41 (s, 2 H), 3.56 (dd, J = 7.2, 7.2 Hz, 2 H), 3.44 (dd, J = 6.2, 6.2 Hz, 2 H), 3.32 (s, 3 H), 1.93-1.85 (m, 2 H). | C16H16ClNO2S2 | 353.89 | 92.96 | C1\C(=C(/C(N1CCCOC)=O)\c1cccs1)\c1sc(cc1)Cl |
| YC-063 | LCMS (Method 2): [MH+] = 324 at 3.59 min | ¹H NMR (400 MHz, CD3CN): δ 7.63-7.61 (m, 1 H), 7.35 (dd, J = 0.4, 2.4 Hz, 1 H), 7.23-7.17 (m, 2 H), 7.02 (d, J = 4.0 Hz, 1 H), 4.45-4.39 (m, 1 H), 4.38 (s, 2 H), 1.29 (d, J = 6.8 Hz, 6 H). | C15H14ClNOS2 | 323.86 | 98.71 | C1\C(=C(/C(N1C(C)C)=O)\c1cccs1)\c1sc(cc1)Cl |
| YC-064 | LCMS (Method 2): [MH+] = 284 at 2.82 min | ¹H NMR (400 MHz, CD3CN): δ 7.20 (dd, J = 1.3, 4.8 Hz, 1 H), 7.12 (d, J = 3.8 Hz, 1 H), 7.01-6.97 (m, 3 H). NH not observed. | C11H6ClNO2S2 | 283.75 | 98.94 | N1\C(=C(/C(O1)=O)\c1cccs1)\c1sc(cc1)Cl |
| YC-065 | LCMS (Method 1): [MH+] = 298 at 3.84 min | ¹H NMR (400 MHz, CD3CN): δ 7.40 (dd, J = 1.2, 5.2 Hz, 1 H), 7.35 (dd, J = 1.2, 3.8 Hz, 1 H), 7.32 (d, J = 4.1 Hz, 1 H), 7.21 (d, J = 4.1 Hz, 1 H), 7.07 (dd, J = 3.8, 5.2 Hz, 1 H), 3.32 (s, 3 H). | C12H8ClNO2S2 | 297.78 | 99.38 | N1(\C(=C(/C(O1)=O)\c1cccs1)\c1sc(cc1)Cl)C |
| YC-066 | LCMS (Method 2): [MH+] = 380 at 3.48 min | ¹H NMR (400 MHz, CD3CN) d 7.62 (d, J=5.1 Hz, 1H), 7.35 (d, J=3.6 Hz, 1H), 7.21 - 7.17 (m, 2H), 7.02 (d, J=4.0 Hz, 1H), 4.41 (s, 2H), 3.93 (dd, J=2.6, 11.4 Hz, 2H), 3.41 - 3.32 (m, 4H), 1.63 (dd, J=1.8, 13.0 Hz, 2H), 1.37 - 1.22 (m, 3H). | C18H18ClNO2S2 | 379.93 | 92.24 | C1\C(=C(/C(N1CC1CCOCC1)=O)\c1cccs1)\c1sc(cc1)Cl |
| YC-067 | LCMS (Method 1): [MH+] = 374 at 3.97 min | ¹H NMR (400 MHz, CD3CN): δ 7.42-7.37 (m, 5 H), 7.33-7.28 (m, 3 H), 7.23 (d, J = 4.0 Hz, 1 H), 7.05 (dd, J = 3.7, 5.2 Hz, 1 H), 4.78 (s, 2 H). | C18H12ClNO2S2 | 373.88 | 94.2 | N1(\C(=C(/C(O1)=O)\c1cccs1)\c1sc(cc1)Cl)Cc1ccccc1 |
| YC-068 | LCMS (Method 1): [MH+] = 295 at 3.55 min | ¹H NMR (400 MHz, CD3CN) d 7.60 - 7.53 (m, 1H), 7.41 - 7.25 (m, 3H), 7.21 (d, J=4.0 Hz, 1H), 7.02 (d, J=4.0 Hz, 1H), 5.29 (s, 2H). | C14H8ClFO2S | 294.73 | 97.18 | C1\C(=C(/C(O1)=O)\c1ccccc1F)\c1sc(cc1)Cl |
| YC-069 | LCMS (Method 1): [MH+] = 338 at 3.87 min | ¹H NMR (400 MHz, CD3CN) d 7.40 (dd, J=1.2, 5.1 Hz, 1H), 7.36 (dd, J=1.2, 3.6 Hz, 1H), 7.33 (d, J=3.7 Hz, 1H), 7.20 (d, J=3.8 Hz, 1H), 7.07 (dd, J=3.5, 5.3 Hz, 1H), 3.52 (d, J=7.1 Hz, 2H), 1.14 - 1.04 (m, 1H), 0.61 - 0.53 (m, 2H), 0.27 - 0.21 (m, 2H). | C15H12ClNO2S2 | 337.85 | 99.5 | N1(\C(=C(/C(O1)=O)\c1cccs1)\c1sc(cc1)Cl)CC1CC1 |

Method 1: UPLC-MS was performed on a UPLC Acquity with AcquityPDA Detector coupled to a Micromass ZQ, Acquity QDA detector single quadrupole mass spectrometer using a Waters BEH column C18 (1.7 µm, 50 x 2.1mm) with a linear gradient of 1-99.9 % acetonitrile/water (with 0.05% formic acid in each mobile phase) within 4 minutes and held at 99.9% for 3.5 minutes; F=1 ml/min.

Method 2: UPLC-MS was performed on a Waters Acquity I-Class with Waters Diode Array Detector coupled to a Waters SQD2 single quadrupole mass spectrometer using an Waters BEH Shield RP18 column (1.7 µm, 100 × 2.1 mm) being initially held at 5% acetonitrile/water (with 10 mM ammonium bicarbonate in each mobile phase) for 1.2 minutes, followed by a linear gradient of 5-100% within 3.5 minutes and then held at 100% for 1.5 minutes (F = 0.5 mL/min).

*SMILES: the simplified molecular-input line-entry system.
